# Marginated aberrant red blood cells induce pathologic vascular stress fluctuations in a computational model of hematologic disorders

**DOI:** 10.1126/sciadv.adj6423

**Published:** 2023-11-29

**Authors:** Xiaopo Cheng, Christina Caruso, Wilbur A. Lam, Michael D. Graham

**Affiliations:** ^1^ Department of Chemical and Biological Engineering, University of Wisconsin-Madison, Madison, WI 53706, USA.; ^2^ Aflac Cancer and Blood Disorders Center of Children’s Healthcare of Atlanta, Department of Pediatrics, Emory University School of Medicine, Atlanta, GA 30307, USA.; ^3^ Wallace H. Coulter Department of Biomedical Engineering. Georgia Institute of Technology and Emory University, Atlanta, GA 30332, USA.

## Abstract

Red blood cell (RBC) disorders such as sickle cell disease affect billions worldwide. While much attention focuses on altered properties of aberrant RBCs and corresponding hemodynamic changes, RBC disorders are also associated with vascular dysfunction, whose origin remains unclear and which provoke severe consequences including stroke. Little research has explored whether biophysical alterations of RBCs affect vascular function. We use a detailed computational model of blood that enables characterization of cell distributions and vascular stresses in blood disorders and compare simulation results with experimental observations. Aberrant RBCs, with their smaller size and higher stiffness, concentrate near vessel walls (marginate) because of contrasts in physical properties relative to normal cells. In a curved channel exemplifying the geometric complexity of the microcirculation, these cells distribute heterogeneously, indicating the importance of geometry. Marginated cells generate large transient stress fluctuations on vessel walls, indicating a mechanism for the observed vascular inflammation.

## Teaser

In blood disorders, aberrant red blood cells drive stress fluctuations at blood vessel walls leading to endothelial inflammation.

## INTRODUCTION

Disorders that affect red blood cells (RBCs) encompass a diverse range of conditions with substantial implications for human health, highlighting the need to comprehend their underlying mechanisms and effects. One prominent example is sickle cell disease (SCD), a monogenic hemoglobin disorder resulting in stiffened and sickle-shaped or otherwise deformed RBCs. SCD leads to chronic pain, organ damage, and life-threatening complications (*1*). In addition, the recent global pandemic of COVID-19, caused by severe acute respiratory syndrome coronavirus 2 infection, can lead to substantial alterations of RBCs (*2*). Emerging evidence suggests that organ dysfunction associated with severe COVID-19 may result from endothelial damage and microvascular thrombosis (*3*). Sepsis, a life-threatening condition arising from severe systemic infection, disrupts the morphology and function of RBCs, precipitating organ failure, hypotension, and increased mortality rates (*4*). Iron deficiency anemia (IDA) adversely affects RBC production and their oxygen-carrying capacity, consequently exerting detrimental effects on the cardiovascular system (*5*). Hereditary spherocytosis, which is characterized by abnormal spherical-shaped RBC, affects cell membrane stability and increases susceptibility to hemolysis (*6*). A comprehensive exploration of these RBC disorders will enable a deeper understanding of their intricacies and the crucial significance of maintaining RBC health and functionality and alleviating vasculopathy.

In individuals with blood disorders, endothelial cells lining the blood vessels are often dysfunctional and, in a proinflammatory state, increase the risk of stroke and atherosclerosis (*7*–*11*). In particular, stroke, a predominant cause of mortality in SCD, often occurs in highly tortuous cerebral arteries and is associated with endothelial inflammation and chronic vasculopathy. In patients with cardiovascular disease and IDA, improved disease outcomes were observed with iron supplementation and subsequent resolution of IDA (*12*); however, the underlying pathophysiologic basis for the association remains unknown. The interplay among adhesive RBC-endothelial interactions, inflammatory cytokines, and hemolysis all contribute to vasculopathy in blood disorders; however, the potential contribution of the altered physical properties of aberrant RBCs, particularly shape and stiffness, to the hemodynamic environment experienced by the vascular endothelium remains poorly understood. This topic is the focus of the present work.

Vascular geometries contribute to vasculopathy in blood disorders (*13*). The vascular system is composed of diverse geometries, including normal complexities such as curves and bifurcations, as well as pathologic ones such as aneurysms and stenoses, and variations in vascular geometry cause substantial changes in the local shear stress profile during blood flow, which are known to induce endothelial proinflammatory responses (*8*, *10*, *13*, *14*). Leveraging an endothelialized microfluidic model of multiple geometries, Mannino *et al.* (*15*) found that vascular cell adhesion molecule–1 (VCAM-1) and E-selectin expression, biomarkers of endothelial cell dysfunction, significantly correlated with shear stress variation and were most pronounced near bifurcation points. Furthermore, they found that endothelial cells exposed to SCD RBCs exhibited increased endothelial inflammation along the outside wall of the bend in the curved regions of vessels (*16*). These observations indicate that it is essential to understand the role of vascular geometric complexity on endothelial dysfunction in blood disorders.

Aberrant RBCs arising in blood disorders often have very different physical properties compared to healthy RBCs. A typical example is SCD, in which abnormal sickle hemoglobin polymerizes within RBCs upon deoxygenation, creating long fibers that pathologically disrupt cellular architecture (*17*), leading to increased membrane stiffness and loss of cellular volume via dehydration. Subsequently, sickle RBCs are biophysically less deformable than normal cells, and some subpopulations are distorted irreversibly into a sickle-like shape. Similarly, in samples of blood from IDA patients, Caruso *et al.* (*18*) identified a subpopulation of very small and poorly deformable iron deficiency RBCs (idRBCs). When exposed to COVID-19, morphologically normal RBCs exhibited a conformational change to sphero-echinocytes with reduced size and deformability (*2*). Relatedly, plasma from adult patients with COVID-19 causes substantial RBC aggregation under flow, and fibrinogen-mediated aggregation directly damages the endothelial glycocalyx (*3*). In hereditary spherocytosis (HS), genetic mutations affect RBC membrane proteins, breaking the linkage between the membrane skeleton and the lipid bilayer, causing membrane loss (*6*). As a result, instead of being biconcave discoids, RBCs become inflexible spherical cells called spherocytes.

The spatial distribution of the different cellular components of blood is nontrivial and depends on the relative physical properties of the different components. Normal RBCs migrate toward the center of a blood vessel, leaving an RBC-depleted cell-free layer (CFL) near vessel walls. In contrast, white blood cells (WBCs) and platelets tend to reside in these layers, a flow-induced segregation phenomenon called margination (*19*–*21*). Experimental observations of modifications to cell segregation in disease are rare. Observing the flow of suspensions composed of SCD RBC populations of two different densities, Clavería *et al.* (*22*) investigated whether segregation occurs among SCD RBCs flowing in micrometer-sized channels. It is known (*23*) that SCD RBCs with higher density exhibit greater shear modulus and consequently increased rigidity. Clavería et al. found a heterogeneous distribution of SCD RBC according to their density: Low-density SCD RBC population remained closer to the center of the channel, while the densest (i.e., stiffest) cells were segregated toward the walls.

The segregation behavior during blood flow is substantially dictated by the contrasts in the cellular properties, such as shape, size, and deformability, of the various components. Kumar e*t al.* (*24*, *25*) used detailed simulations to probe the effect of rigidity difference in a binary suspension of deformable capsules in shear flow. They found that stiff capsules display substantial margination when they are the dilute component, while flexible capsules tend to enrich around the channel’s centerline. Similarly, in a mixture of large and small capsules, the smaller capsules marginate (*25*). Sinha and Graham (*26*) investigated the flow-induced segregation behavior in binary suspensions of spherical and ellipsoidal capsules in simple shear flow by varying the aspect ratio while keeping constant either the equatorial radius or volume of capsules. Direct simulations with models of blood corroborate these model results (*27*, *28*). A simple theory of margination based on the two key transport mechanisms of cells in flow–cell-cell collisions and hydrodynamic migration of deformable cells away from walls (*29*, *30*) predicts that a subpopulation of rigid particles in a suspension of primarily deformable particles will strongly concentrate at walls during flow (*31*, *32*).

Margination may have particular significance in the context of vasculopathy in blood cell disorders. Endothelial cells are responsible for translating biophysical cues, such as the shear force of the hemodynamic microenvironment, into cellular biological signals (*11*, *33*, *34*). Pathological alterations of such forces promote endothelial activation with the release of proinflammatory signals (*35*–*37*), which contribute to atherosclerotic plaques susceptible to myocardial infarction and strokes (*38*). The fact that vasculopathy pervasively occurs even in the oxygenated conditions in both small and larger vessels demands a new understanding of SCD pathophysiology in the absence of vaso-occlusion, which occurs only under the deoxygenated conditions in the microvessels.

Inspired by advances in the mechanistic understanding of the distribution and segregation behaviors during blood flow and experimental observations of blood disorders during flow (*15*, *16*, *18*, *22*, *39*), we propose a biophysical hypothesis for the pathophysiology of vasculopathy in blood disorders: Diseased cells strongly marginate, residing primarily in the CFL near the vascular walls, resulting in endothelial inflammation by provoking fluctuations in local wall shear stress, which is consistent with the chronic and diffusive nature of vasculopathy in blood cell disorders. Limited computational studies of this hypothesis, for blood flow in straight tubes, have been performed for the cases of SCD (*40*) and IDA (*18*).

The present work uses detailed simulations of a cellular-scale mathematical model of blood flow in small vessels to examine this hypothesis. Several diseases are considered: SCD, IDA, COVID-19, and spherocytosis, in both a simple cylindrical blood vessel geometry and a more geometrically complex serpentine curved tube. The choice of these disease models arose from a number of considerations. On biophysical grounds, all of these disorders result in subpopulations of red cells with altered physical, morphological, and geometric properties. Biologically, they represent a spectrum of disorders that encompass different etiologies, illustrating the generalizability of our findings: SCD arises from a genetic disorder, IDA a nutritional one; COVID-19 is an example of an infectious disease that gives rise to biophysically altered red cell subpopulations, and spherocytosis can arise in genetic or acquired disorders. Not only do the results provide strong and broad-based computational support for our hypothesis, but they also begin to reveal transient aspects of the stress environment experienced by endothelial cells and the strong spatial variations in this environment engendered by a complex flow geometry.

An important and distinctive aspect of the study is its focus on mechanism. Because the study is computational, we can interrogate the results in exquisite detail, revealing, for example, not only the presence, but also the physical origin of a strong localization of aberrant cells in specific regions near the walls of a complex blood vessel. Furthermore, as a computational study, it can also avoid factors such as the broad variability in cell properties, which are inevitable in in vivo studies and hard to avoid even in carefully designed in vitro studies, that can obscure the dominant phenomena.

## RESULTS

### Model summary

We simulate a flowing suspension of RBCs, modeled as deformable fluid-filled elastic capsules, in rigid straight and curved cylindrical tubes with diameter *D* = 40 μm. Unless otherwise stated, all results are for simulations that have been run to a statistically stationary state. For the blood disease cases, RBC suspensions are modeled as binary mixtures of normal RBCs with aberrant RBCs from different blood disorders (e.g., idRBCs, sickle RBCs, sphero-echinocytes, and spherocytes). In the binary suspensions, the number fraction is 0.9 for normal RBCs and 0.1 for aberrant RBCs. This is a simplification, as in any real blood cell population, there will be a distribution of cell properties. A suspension of only normal RBCs, referred to as healthy RBC suspension, is considered as a control. The overall volume fraction (tube hematocrit) is around 20%, consistent with the observed substantial decrease of hematocrit from large vessels to the microcirculation (*41*, *42*). (There is some variation between the cases that we consider here because different cell types have different volumes; what we keep constant between cases are number fraction and number density.) The suspending fluid, blood plasma, is considered incompressible and Newtonian with a viscosity of about η = 1.10 to 1.35 mPa·s. The discoid radius *a* for human RBC is about 4 μm. The RBC membrane in-plane shear elasticity modulus *G* ∼ 2.5 to 6 μN/m. The deformability of a capsule in the pressure-driven flow is measured by the dimensionless capillary number $Ca=\eta {\dot{\gamma }}_{w}a/G$. *Ca* is set to be 1.0 for normal RBCs, which corresponds to ${\dot{\gamma }}_{w}∼1000\;s^{-1}$. Increased membrane stiffness has been identified in a range of blood cell disorders. For example, evidence exists that the membrane shear modulus of a typical sickle cell is approximately four times greater than that of a healthy RBC (*43*). For IDA, measurements indicate the presence of iron-deficient RBCs with stiffness up to 10 times greater (*18*). Subpopulations of RBCs with substantially increased membrane stiffness and smaller dimensions have also been found in COVID-19 (*2*) and spherocytosis (*44*). Consequently, for the present study, the interfacial shear modulus *G* of aberrant cells is taken to be five times that of normal RBCs. Therefore, *Ca* for the aberrant RBCs in our study is at most 0.2 times that for normal RBCs.

The spontaneous shape of the RBC membrane is inhomogeneous. Dupire *et al.* (*45*) showed that an RBC maintains its biconcave shape even during tank-treading and hypothesizes that this effect might come from anisotropic elastic properties or an inhomogeneous natural shape. Fischer (*46*) found that RBCs have “shape memory,” which arises from spatial variations in their natural shape. The choice of the spontaneous shape can strongly affect the stable dynamics of the RBC. Sinha and Graham (*47*) investigate the cell dynamics’ dependence on the membrane’s spontaneous curvature. They found that an oblate spheroidal spontaneous curvature maintains the dimple of the RBC during tank-treading dynamics and exhibits off-shear-plane, tumbling consistent with the experimental observations of Dupire *et al.* (*45*). For a complex structure such as an RBC membrane, it is possible that the natural shape for shear elasticity may differ from that for bending elasticity, so the overall natural shape of an element results from the balance of bending and shear forces. Thus, in this work, the spontaneous shape of RBC bending elasticity is taken to be the oblate spheroid, while the spontaneous shape of RBC shear elasticity is assumed to be the biconcave discoid. Further details are included in Materials and Methods and Supplementary Materials; in particular, we show that the results here are robust against changes in the details of the cell elasticity model.

### Cylindrical blood vessel

**Fig. 1.**
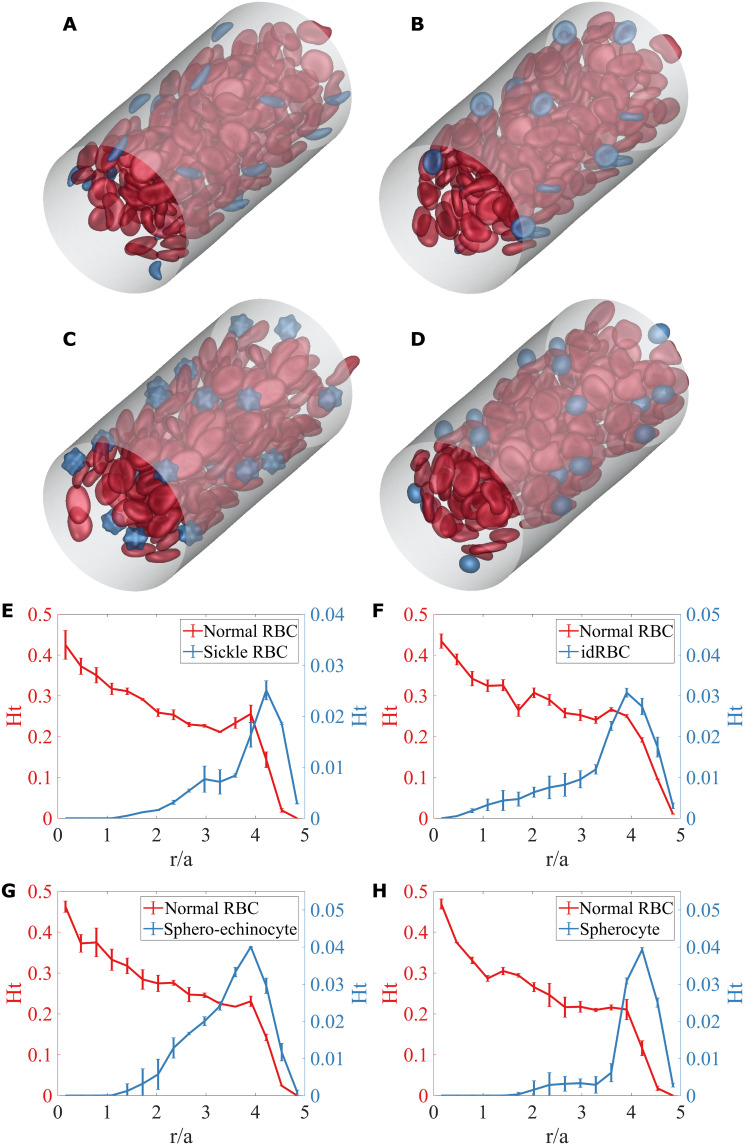
Cell distributions within a cylindrical blood vessel. Top: Simulation snapshots for (**A**) SCD, (**B**) IDA, (**C**) COVID-19, and (**D**) spherocytosis, in straight cylindrical tube subjected to a unidirectional pressure-driven flow. Red capsules represent normal RBCs with oblate spheroid shape for bending elasticity and biconcave discoid shape for shear elasticity, while blue capsules are for aberrant RBCs. Bottom: Radial hematocrit profile for (red) normal RBCs and (blue) aberrant RBCs of (**E**) sickle cells in SCD, (**F**) idRBCs in IDA, (**G**) sphero-echinocytes in COVID-19, and (**H**) spherocytes in spherocytosis. In all figures, error bars represent estimated SE using the block averaging method (*63*).

Figure 1 (A to D) shows snapshots from simulations of blood flow in a straight tube for SCD, IDA, COVID-19, and spherocytosis, respectively. (The Supplementary Materials contain movies of these simulations.) In all cases, the aberrant cells (blue) appear to be marginated. Figure 1 (E to H, respectively) shows the corresponding radial hematocrit profiles. These indicate that aberrant RBCs strongly marginate, while the normal RBCs display the expected CFL and a concentration that increases toward the centerline. Sample simulations with doubled tube length and the same mesh spacing were also conducted; changes in the results were negligible. These results demonstrate that differences in cell size and deformability of the aberrant cells are sufficient to drive strong segregation behavior.

**Fig. 2.**
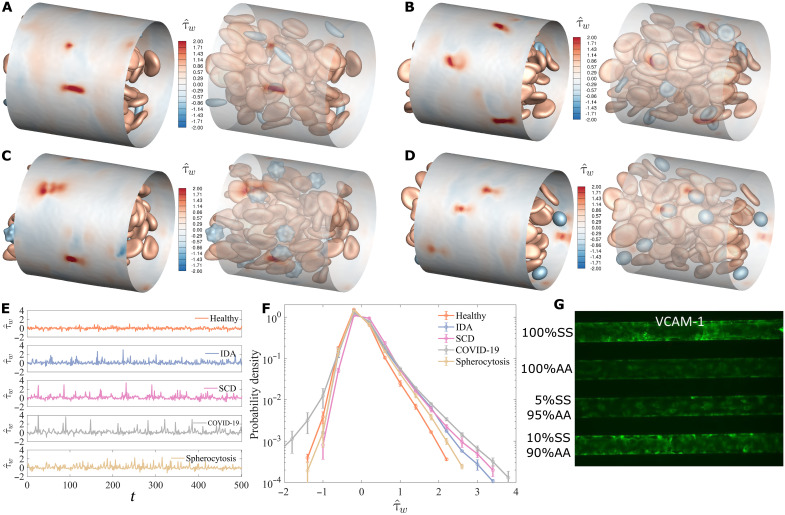
Shear stress on a cylindrical blood vessel. Top: Simulation snapshots and corresponding transparent views of excess wall shear stress ${\hat{\tau }}_{w}$, induced by the presence of the cells in (**A**) SCDs, (**B**) IDA, (**C**) COVID-19, and (**D**) spherocytosis RBC suspensions. To distinguish the colors of the cells themselves from the colors of the RBC-induced wall shear stress on the cylindrical surface, the color of normal RBCs is set to be pale red, and aberrant RBCs are pale blue. Bottom: (**E**) Time evolution of the additional wall shear stress ${\hat{\tau }}_{w}$ (i.e., deviation from the mean) at a fixed wall position for the cases of a homogeneous suspension of healthy RBCs and binary suspensions of normal RBCs with aberrant RBCs, respectively. (**F**) Probability distribution of the additional wall shear stresses ${\hat{\tau }}_{w}$ over the cylindrical wall in the various cases. (**G**) Endothelial VCAM-1 expression in “endothelialized” microfluidic devices with a width of 100 μm after perfusion of suspensions with various fractions of (AA) normal and (SS) SCD RBCs (*39*).

The presence of stiff and/or small aberrant cells near vessel walls is expected to generate high-velocity gradients and, consequently, large shear stresses on the walls, τ*_w_*. Figure 2 (A to D) shows snapshots of the spatial distribution of excess wall shear stress ${\hat{\tau }}_{w}$ for the four cases. Here, ${\hat{\tau }}_{w}=\tau_{w}-{\bar{\tau }}_{w}$ is defined as deviation from the mean wall shear stress ${\bar{\tau }}_{w}$. The red regions indicate large local fluctuations, and one can see that these are directly associated with nearby aberrant RBCs. Figure 2E shows the time series of additional wall shear stress ${\hat{\tau }}_{w}$ at a point on the wall for the various cases. Peaks of high additional wall shear stress are larger and more frequent in all of the disease cases than in the healthy case. These differences are further quantified in Fig. 2F, which shows the probability density profiles of excess wall shear stress in the suspensions. The PDFs for all aberrant RBC cases display a long tail at high ${\hat{\tau }}_{w}$, where the probability density of high wall shear stress for cases with aberrant RBCs is orders of magnitude higher than for the healthy case. This phenomenon is especially prominent for sphero-echinocytes and sickle RBCs, is less pronounced with spherocytes, and is related to their morphology: The spiked surfaces of sphero-echinocytes and sickle RBCs induce high local wall shear stress, while the round spherocytes, although near the wall, roll smoothly without generating substantial excess stress.

The simulation findings here are further compared with previous experimental observations in Fig. 2G. To investigate the role of cellular interaction in hematological diseases, Caruso *et al.* (*39*) developed an in vitro microvasculature model composed of endothelial cells cultured through the inner surface of a microfluidic system. SCD RBCs were spiked into normal RBC suspension, suspended, and then perfused into this endothelialized microfluidics. They found that VCAM-1, a biomarker of endothelial cell dysfunction, was up-regulated when exposed to flowing SCD RBCs than normal RBCs. These results together imply that purely physical interactions between endothelial cells and SCD RBCs are sufficient to cause endothelial inflammation.

Moreover, recent research further corroborates our current observations. Specifically, in the context of diabetes-associated oxidative stress leading to reduced RBC deformability, Czaja *et al.* (*48*) used simulations to investigate pulsatile blood flow through segmented retinal microaneurysms. Their findings revealed that diabetic RBCs, characterized by increased stiffness, induced higher local wall stress and wall shear stress gradients within leading and draining parental vessels, compared to their healthy RBC counterparts. In addition, leveraging a high-fidelity computational model of blood flow, Ebrahimi and Bagchi (*49*) revealed that reduced cell deformability causes substantial changes in microvascular hemodynamics, and alteration in RBC dynamics induces localized changes in wall shear stress within vessels and in proximity to vascular bifurcations. However, these investigations, while addressing the influence of blood cell deformability on hemodynamics and wall shear stress, did not incorporate the RBC margination driven by deformability difference as a potential contributor to vascular wall stress fluctuations.

### Curved blood vessel

**Fig. 3.**
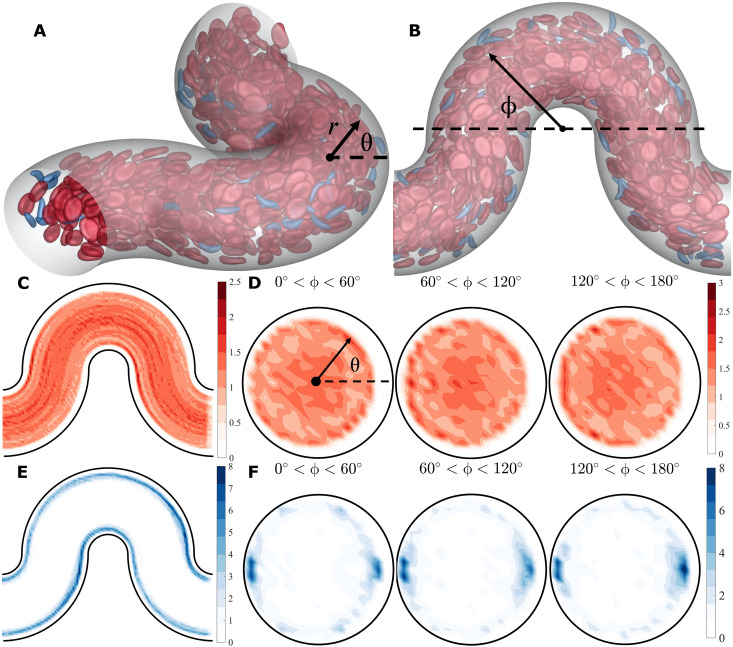
Cell distributions within a curved blood vessel. (**A** and **B**) Simulation snapshots of a suspension of normal RBCs with sickle RBCs in a curved channel, showing coordinate definitions for *r*, the distance from the local channel centerline, θ, the relative position between the “inner side” (θ = 180°) and “outer side” (θ = 0°), and ϕ, the angle around the curve. (**C**) Normalized center-plane cell number density and (**D**) normalized cross-sectional cell number density for normal RBCs in the binary SCD RBC suspension. (**E**) Normalized center-plane cell number density and (**F**) normalized cross-sectional cell number density for sickle RBCs in the binary SCD RBC suspension. Note that the curved channel is divided into three parts on the basis of the value of angle ϕ; thus, the cross-sectional cell number distribution is computed over each part. The cell number density distributions are normalized so that if the spatial distributions of cells are uniform, then the normalized cell number density is unity everywhere within the curved channel.

We now consider cell and stress distributions in a curved tube. Figure 3 presents simulation results for a suspension of normal RBCs with sickle RBCs. Snapshots of cell distributions are shown in Fig. 3 (A and B), along with a coordinate system that we use for the analysis. Figure 3 (C and E) show cell number density distributions for the (C) normal and (E) aberrant cells on the center plane of the channel. The margination of the aberrant cells is apparent. Figure 3 (D and F) show the number density distributions for the normal and aberrant cells averaged over various segments of the channel, including both the normalized center-plane and cross-sectional cell distributions. Figure 3D shows that the CFL thickness is larger near the outer side (θ = 0°) and thinnest near the inner side (θ = 180°). Figure 3F indicates that sickle RBCs strongly focus at two near-wall locations, both at the inner and outer sides, on the center plane. As the angle ϕ increases (i.e., as we move downstream around a bend), the concentration of sickle cells near the outer wall becomes more pronounced. Similar results are found for the other aberrant cell suspensions as well, as seen in the cross-sectional and center-plane distributions shown in fig. S11.

**Fig. 4.**
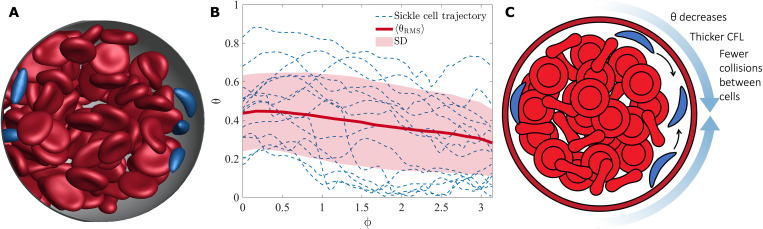
Mechanism of cell localization on the symmetry plane of a curved vessel. (**A**) Simulation snapshot showing the cross-sectional cell distribution (ϕ = π/2). (**B**) Trajectories of sickle RBCs on the θ-ϕ plane. Dashed blue curves are individual trajectories; the red line denotes the RMS trajectory of the blue curves, and the red shaded area is for the corresponding SD. (**C**) A schematic mechanism for localization of marginated cells to θ = 0.

These results demonstrate that in the curved tube, we see not only margination of aberrant cells as found in the straight tube but also very strong localization of the marginated cells on the center plane. The mechanism of this localization originates in the θ-dependence of the CFL thickness, as illustrated in Fig. 4. Figure 4A shows a simulation snapshot of the cross-sectional cell distribution at ϕ = π/2, in which aberrant cells are highly localized near the outer side center plane. We noted above that the CFL thickness is approximately uniform for π/2 < θ < 3π/2, i.e., along the inner wall, but on the outer side, the CFL thickness increases, reaching a maximum on the center plane on the outer wall, i.e., the CFL thickness increases as θ → 0. A marginated cell on the outer wall will experience more collisions from the side with the thinner CFL than the thicker, thus being driven, on average, toward the region where the CFL is the thickest, θ = 0. Figure 4B shows the trajectories of marginated aberrant RBCs on the θ − ϕ plane, demonstrating that as ϕ increases, θ tends to decrease and aberrant cells move toward the center plane. We illustrate this mechanism schematically in Fig. 4C. Last, we must address why there is localization along the center plane on the inner wall (θ = π). This results from the simple fact that the outer wall over half a wavelength of the curved shape is the inner wall over the other half; the CFL thickness is nearly constant along the inner wall, driving no net motion in θ, and cells driven toward θ = 0 on the outer wall tend to remain there while moving along the inner wall.

**Fig. 5.**
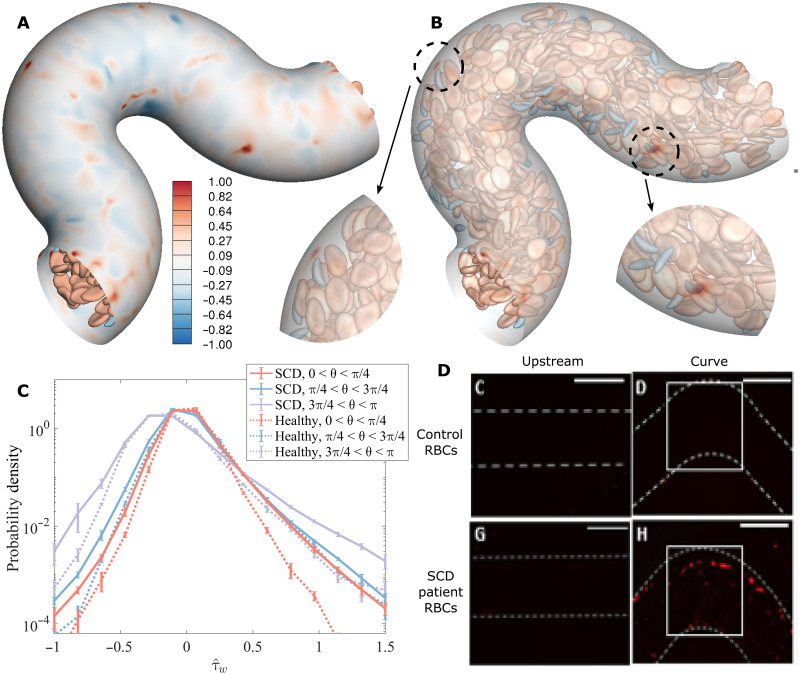
Shear stress on a curved blood vessel. (**A**) Simulation snapshots and (**B**) corresponding transparent views of additional wall shear stress ${\hat{\tau }}_{w}$ induced by the presence of the cells in suspensions of normal RBCs with sickle RBCs within the curved channel. The color on the vascular surface denotes the RBC-induced wall shear stress strength ${\hat{\tau }}_{w}$. (**C**) The time-averaged probability distribution of the additional wall shear stress ${\hat{\tau }}_{w}$ for SCD RBC suspension and healthy RBC suspension over the different θ area on the curved vascular surface. (**D**) Mechanically stiff SCD RBCs up-regulate E-selectin at the curvature site of vasculature models, indicating proinflammatory endothelial signaling (*16*). Scale bars, 200μm.

Figure 5A shows snapshots of the spatial distribution of excess wall shear stress ${\hat{\tau }}_{w}$ in SCD suspensions; results for the other blood disorders can be found in figs. S12 to S14. The presence of a sickle RBC close to the wall directly causes local fluctuations in wall shear stress, as can be observed from the transparent view in Fig. 5B.

To capture the spatial dependence of RBC-induced wall shear stress ${\hat{\tau }}_{w}$, the probability density profiles of τ*_w_* in SCD and healthy RBC suspensions over the different θ areas on the vascular surface are presented in Fig. 5C. In the healthy RBC suspension, the probability of high ${\hat{\tau }}_{w}$ is largest near the inner side (3π/4 < θ < π), followed by the intermediate area of (π/4 < θ < 3π/4), and smallest over the outer side (0 < θ < π/4), consistent with the fact that the CFL is thinnest near the inner side and thickest near the outer side. As in the straight tube, it is observed that the cases with aberrant RBCs exhibit a distinct excess of large positive fluctuations, again attributable to the margination of these small stiff cells to the vessel wall. Furthermore, the disparity at high RBC-induced wall shear stress ${\hat{\tau }}_{w}$ of the probability distribution profiles between diseased and healthy RBC suspensions is most pronounced at the outer side, which implies that the localization of the marginated aberrant RBCs to the center plane elevates the probability of high additional wall shear stress by an order of magnitude over the outer side wall of the curved tube.

The geometries of blood vessels have been found to play a role in the development of endothelial dysfunction in blood disorders. Using an endothelialized microfluidic model, Wang *et al.* (*16*) discovered that endothelial cells exposed to SCD RBCs exhibited an increase in endothelial inflammation along the outside wall of the bend in the curved regions of vessels. This result is illustrated in Fig. 5D, which shows expression levels of E-selectin, which is up-regulated when cells are in a proinflammatory state, upstream, and in a curved segment of endothelialized microfluidic channels through which RBC suspensions have flowed. While the channel sizes in the experiments are much larger than those simulated here, the qualitative pattern of endothelial inflammation there is consistent with the margination patterns that we observe here.

**Fig. 6.**
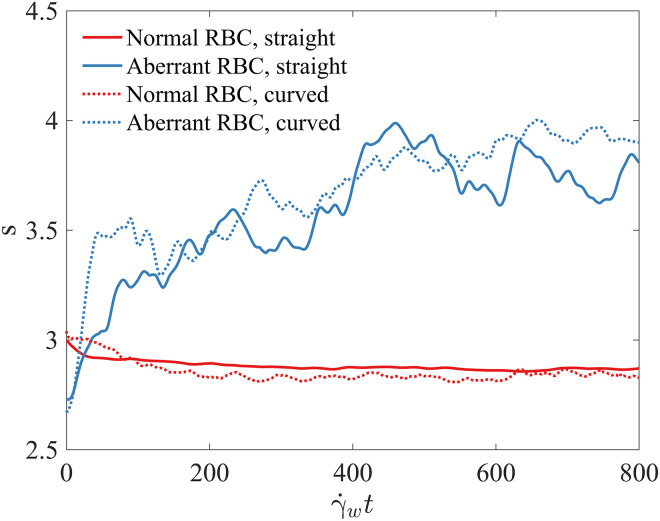
Segregation in straight and curved channels. Temporal evolution of RMS distance from the centerline, $s={\left\langle r_{\mathrm{cm}}^{2}\right\rangle }^{1/2}/a$ for each cellular component (red, normal RBCs; blue, aberrant RBCs) in an SCD RBC suspension within the straight tube (solid line) and curved channel (dash line). *r_cm_* is the radial center-of-mass position of a cell, and angle brackets denote averaging over the cells in the system. Both the cylindrical straight and curved channels have a tube radius of 20 μm.

To further quantify the cellular segregation in different geometries, we measure the scaled root mean square (RMS) distance from the centerline, $s={\left\langle r_{\mathrm{cm}}^{2}\right\rangle }^{1/2}/a$, for each cellular component in straight and curved channels. At the start of the simulations, all cells are randomly distributed within the vascular channels. Figure 6 illustrates the temporal evolution of *s* for both normal and sickle RBCs. Initially, the aberrant RBCs show a sharp increase in *s* and then plateau with *s* ≈ 3.8; in contrast, for normal cells, *s* ≈ 2.9. This behavior is similar in both straight and curved channels, consistent with the above observations that aberrant cells are localized to vessel walls. Here, we have measured time in terms of strain units ${\dot{\gamma }}_{w}t$, and Fig. 6 indicates that, for margination under the present conditions, ∼200 strain units are required for margination. This can be translated roughly into downstream distance by noting that the mean velocity for laminar flow with wall shear rate ${\dot{\gamma }}_{w}$ is ${\dot{\gamma }}_{w}R$. Thus, in the time of about 200 strain units required for margination to take place, a cell moving at the mean velocity travels 2 mm.

In addition, we observe that segregation occurs somewhat more rapidly in the curved channel compared to the straight channel, as indicated by the faster growth of *s* for aberrant cells after the simulation starts. While we are unaware of other work on margination in serpentine geometries, relatedly, Bächer *et al.* (*50*) explored the margination of microparticles in blood flow through bifurcations within vascular networks. They found that bifurcations did not substantially influence the margination propensity of stiff particles relative to flow in straight tubes.

## DISCUSSION

Blood disorders lead to changes in RBC size, shape, and stiffness and, thus, to changes in how aberrant cells are distributed in the cross section of blood vessels and changes in the interaction between cells and blood vessels. Inflammation and dysfunction of endothelial cells lining blood vessels are associated with the risk of pathophysiologic complications like stroke and atherosclerosis.

This study describes results from detailed cell-level simulations of blood in straight and serpentine tubes, addressing the hypothesis that the margination of aberrant cells leads to substantial changes in the local shear stress environment of the blood vessel wall, possibly contributing to the observed dysfunction and inflammation. We compare cell distributions and wall shear stress profiles between suspensions of normal blood and blood containing aberrant RBCs that model SCD, IDA, COVID-19, and spherocytosis. In all cases, the smaller and stiffer aberrant RBCs marginate and increase the fluctuations in wall shear stress. Probability density profiles of wall shear stress show that cases with aberrant RBCs display a significantly higher probability of high wall shear stress than in suspensions of healthy cells. The difference is most notable in COVID-19 and SCD RBC suspensions.

In the serpentine curved tube case, the marginated aberrant cells tend to marginate most strongly to the symmetry plane of the channel and the outer side of the curved tube, becoming very strongly localized in those regions. This result implies the possibility of strongly localized endothelial damage in blood vessels with complex geometries. These findings highlight the importance of considering vascular geometry and the presence of aberrant RBCs in the development of vasculopathy.

Overall, our study indicates that the biophysical alterations of red cells in various disorders, in and of themselves, can directly alter the shear stress the underlying endothelium is exposed to. This suggests that that red cell biophysics and the pathologic changes thereof may directly affect endothelial mechanobiological pathways, which, in turn, may be associated with chronic endothelial inflammation or dysfunction that lead to disorders such cardiovascular disease and stroke. Experiments to complement these computational results are now being conducted in our group. In addition, our study also suggests that, clinically, more attention should be paid to the biophysical alterations of the red cells themselves, which currently are viewed as hallmarks of the associated disease but not necessarily as biomarkers per se; our work suggests otherwise and indicates that the red cell shape, size, and deformability should be reexamined more rigorously as potential correlates or predictors of clinical end points. Last, our work indicates that therapeutic interventions that “fix” or remove the biophysical alterations of red cells should be explored for the associated diseases. Red cell pheresis for various hematology conditions may improve the vascular dysfunction associated with those diseases, and for SCD in particular, recently, Food and Drug Administration–approved treatments that improve “red cell health” may also alleviate vasculopathy.

## MATERIALS AND METHODS

We consider a flowing suspension of RBCs, which we model as deformable fluid-filled elastic capsules, in rigid straight (Fig. 1) and curved “serpentine” (Fig. 3) cylindrical tubes with radius *R* = 20 μm. No-slip boundary conditions are imposed on the walls of the tube, while periodic boundary conditions are applied in the flow direction. The suspension is subjected to a unidirectional pressure-driven flow, and the velocity field in the absence of RBCs field within the straight cylindrical tube is given by the Poiseuille flow. In this study, the flow is driven by a constant pressure gradient, which is equivalent to fixing the mean wall shear rate at ${\dot{\gamma }}_{w}=2U_{0}/R$, where *U*_0_ is the undisturbed centerline velocity. For the curved cylindrical channel, the pressure gradients are determined by defining an equivalent straight cylindrical channel with the same centerline length and radius.

This study considers both homogeneous and heterogeneous suspensions of various components, including normal RBCs and aberrant RBCs (e.g., idRBCs, sickle RBCs, sphero-echinocytes, and spherocytes). In binary suspensions, normal RBCs are generally considered primary components (denoted as “p”), while aberrant RBCs are considered as trace components (denoted as “t”). A normal RBC is modeled as a flexible capsule having the spontaneous shape being a biconcave discoidal for shear elasticity and an oblate spheroid for bending elasticity (*47*, *51*), with a radius of *a* = 4 μm. The idRBCs have the same rest shape as normal RBCs, except that the radius of idRBCs is 0.76*a* (*18*). The rest shapes of sickle RBCs, spherocytes, and sphero-echinocytes are curved oblate, spherical with a diameter of 5 μm, and spiked spherical, respectively. The cell membranes are modeled as an isotropic and hyperelastic surface with interfacial shear modulus *G*, incorporating shear elasticity, area dilatation, volume conservation, and bending resistance. Details of the membrane mechanics model and validation against experimental observations are given in (*47*).

The deformability of a capsule in pressure-driven flow is measured by the dimensionless capillary number $Ca=\eta {\dot{\gamma }}_{w}a/G$. In this study, *G* of the aberrant RBCs is assumed to be five times that of normal RBCs, which leads to that of *Ca*_t_ for aberrant RBCs is always around less 0.2 times that of *Ca*_p_ for biconcave discoid RBCs. In this study, *C*a_p_ is set to 1.0 for normal RBCs, *C*a*_t_* is 0.15 for idRBCs, 0.20 for sickle RBCs, 0.15 for sphero-echinocytes, and 0.125 for spherocytes, which corresponds to ${\dot{\gamma }}_{w}∼1000\;s^{-1}$. In the binary suspension, the number fractions for normal RBCs *X_b_* is set to 0.9, and for aberrant RBCs, *X_t_* is 0.1, so the overall number density ratio *n_p_*/*n_t_* = 9. In this study, the total cell volume fraction (hematocrit) is set to be ϕ ≈ 0.20. To simplify the computations in this initial study, the suspending fluid and the fluid inside the cells are assumed to have the same viscosity.

In our simulation, the particle Reynolds number, defined as $Re_{p}=\rho {\dot{\gamma }}_{w}a^{2}/\eta$, is set to be 0.1, and the fluid is assumed to be incompressible and Newtonian; therefore, the flow is governed by the Navier-Stokes and continuity equations. A projection method is used to advance the velocity field in time. The straight tube is embedded in a cuboidal computational domain with the size of 10*a* × 10*a* × 10*a*, and an Eulerian grid of 100 × 100 × 100 is used. For the serpentine channel case, the cuboidal computational domain’s size is 32*a* × 26*a* × 10*a*, and the Eulerian grid of 320 × 260 × 100 is used. The immersed boundary method (IBM) is used to handle fluid-structure interaction. Specifically, the current model considers two types of immersed boundaries: deformable moving cellular membranes and rigid nonmoving vascular walls. The capsule membrane is discretized into *N*_Δ_ piecewise flat triangular elements: *N*_Δ*p*_ = 1280 for normal RBC, while *N*_Δ*t*_ = 682 for sickle RBC. *N*_Δ*t*_ = 816 for idRBC. *N*_Δ*t*_ = 620 for spherocytes, and *N*_Δ*t*_ = 1134 for sphero-echinocytes. Different *N*_Δ_ are chosen to ensure that the triangular elements on both capsules are close in size. We use “continuous forcing” IBM and “direct forcing” IBM methods for the RBC membranes and tube wall, respectively. The numerical methodology follows the approach described in (*52*, *53*).

Several limitations pertain to the model that we used in this study. First, we assume a viscosity ratio of 1 between the intercellular matrix and plasma, whereas normal RBCs may exhibit a ratio of up to 15 (*54*), and aberrant RBCs may have even higher ratios. As the RBC ages, the viscosity of the cytosol increases (*55*). Nonetheless, past research (*56*) has demonstrated that the dynamics of a single sickle cell remain qualitatively unchanged over a broad range of viscosity ratio. In addition, Reasor *et al.* (*57*) performed simulations to investigate the margination dependence on the viscosity ratio, finding that the cell-free layer thickness and margination of stiff cellular components remain similar. Another limitation of this study is the assumption of uniform shape, size, and stiffness within each red cell subpopulation, which we know does not fully account for the inherent diversity in these properties present in real red cell populations (*58*, *59*). Nevertheless, while the physical properties of blood cells exhibit dispersity, the variation of these properties within one cell subpopulation is substantially narrower when compared to the variation between different cell subpopulations [see, e.g., (*18*)]. Thus, we expect that this simplification does not alter the main conclusions drawn here.

Furthermore, sickle cells display increased adhesiveness to the vascular wall, triggering the vaso-occlusion that is the hallmark of SCD. Clavería *et al.* (*22*) underscore the role of cell aggregation in mitigating segregation and amplifying CFL thickness, potentially guarding against vaso-occlusion in sickle cell anemia patients. In the present work, we do not consider intercellular forces such as aggregation and adhesion, for two reasons. First, our focus is the broader issue of the role of margination across blood disorders, not just SCD. Furthermore, aggregation effects are most prominent at low shear rates, ≲10 s^−1^, whereas the wall shear rate ${\dot{\gamma }}_{w}$ in our simulation remains substantially higher, ∼1000 s^−1^, a condition representative of venules and arterioles.

Last, endothelial cells exposed to shear stress express an endothelial glycocalyx layer (EGL) that plays a central role in mediating vascular permeability and endothelial anti-inflammatory, antithrombotic, and antiangiogenic properties (*60*). Notably, EGL disruption occurs in arterial regions exposed to disturbed blood flow, accompanied by elevated oxidative stress leading to atherogenesis (*61*, *62*). Our model, which treats the vascular surface as a nonslip rigid boundary, does not capture the permeability and porosity characteristics of EGL. However, it is important to underscore that the presence of EGL, while affecting interactions of RBC with vessel walls, is not anticipated to significantly affect the margination of aberrant cells, which primarily arises from contrasting cellular properties between aberrant and normal RBCs. In addition, our study focuses on conditions within venules and arterioles, where vessel-cell interactions are less pronounced compared to the confined spaces of capillaries. Therefore, the limitations of our model do not change the central findings in this research: The aberrant cells strongly marginate, residing primarily in the CFL and generating physical interactions that damage the endothelium.

## Supplementary Materials

### This PDF file includes:

Materials and Methods Supplementary Text Figs. S1 to S14 Legends for movies S1 to S8 References
