# Supplementary material for: Marginated aberrant red blood cells induce pathologic vascular stress fluctuations in a computational model of hematologic disorders
Source: Sci Adv. 2023 Nov 29;9(48):eadj6423. doi: 10.1126/sciadv.adj6423 (PMC10686556; doi:10.1126/sciadv.adj6423)
Supplement: Supplementary file 1 — Materials and Methods Supplementary Text Figs. S1 to S14 Legends for movies S1 to S8 References [file sciadv.adj6423_sm.pdf]

Science Advances
AAAS

Supplementary Materials for

Marginated aberrant red blood cells induce pathologic vascular stress fluctuations in a computational model of hematologic disorders

Xiaopo Cheng *et al.*

Corresponding author: Wilbur A. Lam, wilbur.lam@emory.edu; Michael D. Graham, mdgraham@wisc.edu

Sci. Adv. **9**, eadj6423 (2023)
DOI: 10.1126/sciadv.adj6423

The PDF file includes:

Materials and Methods
Supplementary Text
Figs. S1 to S14
Legends for movies S1 to S8
References

Other Supplementary Material for this manuscript includes the following:

Movies S1 to S8

Materials and Methods

We have developed a computational model utilizing the immersed boundary method (IBM) to investigate cellular blood flow in complex vessel geometries. This approach offers the advantage of modeling flows in arbitrary geometries and accurately resolving the large deformation and dynamics of blood cells. Our model considers blood as a confined flowing suspension of red blood cells (RBCs) in the plasma. Two distinct types of boundaries are involved: deformable cellular membranes and rigid non-moving vascular walls with complex geometry. To handle these two types of interfaces, we use the continuous-forcing and direct-forcing immersed boundary methods, respectively. Specifically, the continuous-forcing IBM method couples surface stresses on the RBC membrane with the fluid flow, while the sharp-interface ghost-node immersed boundary method (GNIBM) is used to treat the rigid, non-moving vascular walls (*52, 53*). The flow solver is based on a coupled finite-volume/spectral method, and our IBM code is parallelized using a hybrid MPI/OpenMP strategy.

Governing Equations

Assuming that blood plasma is both Newtonian and incompressible, the governing equations for its flow can be expressed as the incompressible Navier-Stokes equations.

$$\begin{aligned} Re\left(\frac{\partial \boldsymbol{u}}{\partial t} + \nabla\cdot\boldsymbol{u}\boldsymbol{u}\right) &= -\nabla P + \nabla^2\boldsymbol{u} + \boldsymbol{F} \\ \nabla\cdot\boldsymbol{u} &= 0 \end{aligned} \tag{1}$$

Flow Solver

The Chorin projection method is utilized to advance the velocity field $\boldsymbol{u}$. This method involves solving an advection-diffusion equation (ADE) to determine the intermediate velocity field $\boldsymbol{u}^*$, followed by solving a Poisson equation for pressure P to enforce the divergence-free

constraints.

$$
\begin{aligned}
Re\frac{\boldsymbol{u}^* - \boldsymbol{u}^n}{\Delta t} &= \nabla^2 \boldsymbol{u} - Re\nabla \cdot \boldsymbol{u}\boldsymbol{u} + \boldsymbol{F} \\
\nabla^2 P &= \frac{Re}{\Delta t}\nabla \cdot \boldsymbol{u}^* \\
\frac{\boldsymbol{u}^{n+1} - \boldsymbol{u}^*}{\Delta t} &= -\frac{1}{Re}\nabla P
\end{aligned} \tag{2}
$$

For the ADE, we treat both the convection term and the body force term with the 2nd-order Adams-Bashforth method (AB2), and the diffusion term with the Crank-Nicholson method for numerical stability.

$$
Re\frac{\boldsymbol{u}^* - \boldsymbol{u}^n}{\Delta t} = 0.5\nabla^2(\boldsymbol{u}^* + \boldsymbol{u}^n) + 1.5N\left(\boldsymbol{u}^n\right) - 0.5N\left(\boldsymbol{u}^{n-1}\right) + 1.5\boldsymbol{F}^n - 0.5\boldsymbol{F}^{n-1} \tag{3}
$$

where $N(\boldsymbol{u}) = -Re\nabla \cdot \boldsymbol{u}\boldsymbol{u}$ denotes the nonlinear convection, evaluated then by the central differencing. To leverage the efficient inversion of tri-diagonal matrices, a Locally One-Dimensional (LOD) Alternating Direction Implicit (ADI) scheme is employed to solve the ADE. This scheme involves four steps: first, the explicit terms are handled; then, the x, y, and z directions are solved implicitly, one at a time. To solve the 3D pressure Poisson equation (PPE), we assume that the computational domain is periodic in two directions, namely x and y. We begin by performing a 2D fast Fourier transform (FFT) on each $x - y$ plane. Next, solve for the Fourier coefficients along the z direction. Finally, perform a 2D inverse fast Fourier transform (iFFT) on each $x - y$ plane to obtain the pressure field.

Spatial Discretization

The IBM requires an Eulerian representation for the fluid flow and a Lagrangian representation for the immersed boundaries such as the RBC membrane. The governing equations are solved on the Eulerian grid. To avoid odd-even decoupling, spatial discretization is based on the staggered mesh, in which these scalar variables, such as pressure P, are located at the grid center, while vector variables, such as velocity u and force F, are located at the grid faces. All spatial derivatives are evaluated using second-order differencing. As for the Lagrangian mesh on the

immersed boundaries, the membrane is discretized into piecewise flat triangular elements. An open-source software Gmsh (*64*) is used to generate the surface mesh for both vessels (Fig. **S1.**) and various red blood cells (Fig. **S2.**).

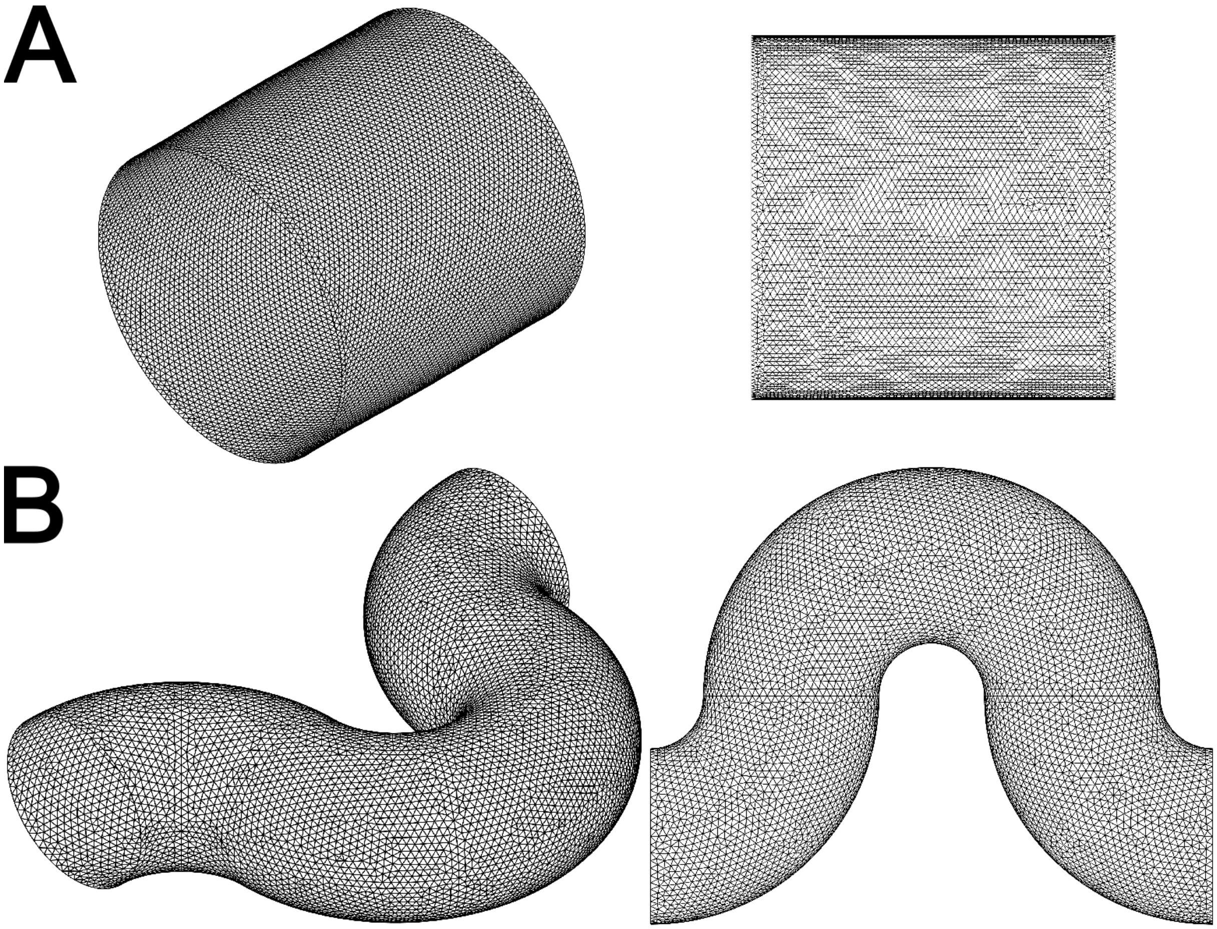

Fig. S1. Surface mesh for (A) a straight cylindrical tube (radius $R = 20\mu m$ and length $L = 40\mu m$) and (B) a curved (serpentine) cylindrical channel (major radius $R_1 = 32\mu m$ and minor radius $R_2 = 20\mu m$).

Membrane Mechanics

The RBC membrane is assumed to resist shear deformation, area dilatation, volume conservation (*65*), and bending resistance. The total strain energy of the RBC membrane S is given

by,

$$E = \frac{K_B}{2} \int_S (2\kappa_H + c_0)^2 \, dS + \overline{K_B} \int_S \kappa_G dS + \int_S W dS$$

where K_B and $\overline{K_B}$ are the bending moduli, and W is the shear strain energy density; κ_H, κ_G are the mean and Gaussian curvature of the surface, respectively; $c_0 = -2H_0$ is the spontaneous curvature, where H_0 is the mean curvature of the spontaneous shape. The first two terms correspond to the Canham-Helfrich bending energy (*66,67*), and the third term comes from the shear strain energy stored in the RBC membrane. The strain energy density is computed based on the Skalak model (*68*). A finite element method (FEM) is developed to find the surface stress as a result of membrane deformation (*47*).

Continuous Forcing IBM

The cellular membrane is deformed by the fluid flow, while the flow is altered by the membrane deformation in turn. This fluid-structure interaction (FSI) between flow and membrane is characterized via the continuous forcing IBM. The idea of this method is to add an external force term $\boldsymbol{F}$ to the right-hand side of the Navier-Stokes equation. The external force term originates from the membrane stress f_{membrane}. A discretized delta function is used to spread the singular force on the membrane to the surrounding fluid and interpolate the fluid velocity back to the membrane.

$$\begin{aligned} \boldsymbol{F} &= \int_S \boldsymbol{f}_{\text{membrane}} \, \delta\left(\boldsymbol{x} - \boldsymbol{x}'\right) d\boldsymbol{x}' \\ \boldsymbol{u}_{\text{membrane}} &= \int_{\mathcal{T}} \boldsymbol{u} \delta\left(\boldsymbol{x}' - \boldsymbol{x}\right) d\boldsymbol{x} \end{aligned} \tag{4}$$

δ is the three-dimensional Dirac-delta function, and $\mathbf{x}$ and $\mathbf{x}'$ are the locations in the flow domain $\mathcal{T}$ and on the cell surface S, respectively. A numerical approximation of the delta function is chosen to be

$$\delta\left(\mathbf{x} - \mathbf{x}'\right) = \begin{cases} \dfrac{1}{64\Delta^3} \displaystyle\prod_{i=1}^{3} \left[1 + \cos\frac{\pi}{2\Delta}\left(x_i - x_i'\right)\right], & |x_i - x_i'| \leq 2\Delta \\ 0, & |x_i - x_i'| > 2\Delta \end{cases} \tag{5}$$

where Δ is the Eulerian grid size.

Direct Forcing IBM

Direct forcing IBM is used to treat another type of immersed boundary, the rigid, non-moving but geometrically complex vessel walls. Specifically, GNIBM is used to impose no-slip velocity boundary conditions on the vessel surface (*53*). The idea of this method is to modify these differential operators appropriately to satisfy the no-slip boundaries condition on the geometrically-complex vascular surface while maintaining second-order accuracy.

Indicator Function for Hematocrit Analysis

To characterize the cellular spatial distribution in RBC suspension, we define an indicator function $I(\mathbf{x}, t)$, such that the indicator function is one inside a cell, and zero outside a cell. It can be shown (*69*) that the indicator function $I(\mathbf{x}, t)$ follows a Poisson equation as

$$\nabla^2 I = \nabla \cdot \mathbf{G}, \quad \mathbf{G}(\mathbf{x}, t) = \int_S \delta\left(\mathbf{x} - \mathbf{x}'\right) \mathbf{n} dS \tag{6}$$

where the $\mathbf{G}(\mathbf{x}, t)$ is an Eulerian variable constructed from the cell surface normals $\mathbf{n}$.

Wall Shear Stress Evaluation

To compute wall shear stress, the traction vector $\mathbf{t} = \boldsymbol{\tau} \cdot \mathbf{n}$ at the wall is determined using the velocity field approach outlined in (*70, 71*), where $\boldsymbol{\tau}$ is the stress tensor and $\boldsymbol{n}$ is the unit normal vector. The no-slip condition on the blood vessel surface yields the expression for the local wall shear stress $t_s = \mu \partial u_s / \partial r$. Note s denotes the local stream-wise direction. The second-order differencing method is utilized to numerically evaluate velocity derivatives.

Model Verification and Validation

Elastic Spherical Capsule in Simple Shear Flow

We consider a moving deformable spherical capsule subjected to simple shear flow, see Fig.**S3.**(A). The deformation of a capsule will cause stretching stress and bending stress on its surface. The deformability of a capsule is characterized by the nondimensional Capillary number $\mathrm{Ca} = \mu\dot{\gamma}a/G$ and the nondimensional bending modulus $\hat{\kappa}_B = K_B/a^2G$, where μ is the fluid viscosity, $\dot{\gamma}$ is shear rate, a is the radius of the capsule, G is the shear elasticity modulus and K_B is the bending modulus. Larger Ca and $\hat{\kappa}_B$ means that a capsule is more flexible, while a stiffer capsule has smaller Ca and $\hat{\kappa}_B$. The deformation of the capsule is described by the Taylor shape parameter defined as $D_{xz} = (L-B)/(L+B)$, where L and B are the maximum and minimum radial distances of an ellipsoid with the same inertia tensor. In Fig.**S3.**(B), we show that steady state values of Taylor deformation parameter D as a function of dimensionless Capillary number Ca for different $\hat{\kappa}_B$. Good agreement is found between our numerical results and simulation results from previous literature (*72*).

Stationary Rigid Sphere in Simple Shear Flow

We consider a rigid stationary sphere subjected to a linear shear flow, which is given by $\mathbf{u}^{\infty} = [\dot{\gamma}z, 0, 0]$. The schematic of the model set is shown in Fig.**S4.**(A). The analytical solution for this problem is given by

$$\begin{aligned} u &= \frac{y\dot{\gamma}}{2}\left[1-\left(\frac{r}{a}\right)^{-5}\right] + \frac{y\dot{\gamma}}{2}\left[1-\left(\frac{r}{a}\right)^{-3}\right] - \frac{5}{2}\left(\frac{x}{a}\right)^{2} y\dot{\gamma}\left[\left(\frac{r}{a}\right)^{-5} - \left(\frac{r}{a}\right)^{-7}\right] \\ v &= \frac{x\dot{\gamma}}{2}\left[1-\left(\frac{r}{a}\right)^{-5}\right] - \frac{x\dot{\gamma}}{2}\left[1-\left(\frac{r}{a}\right)^{-3}\right] - \frac{5}{2}\left(\frac{y}{a}\right)^{2} x\dot{\gamma}\left[\left(\frac{r}{a}\right)^{-5} - \left(\frac{r}{a}\right)^{-7}\right] \\ w &= -\frac{5}{2}\frac{xyz\dot{\gamma}}{a^2}\left[\left(\frac{r}{a}\right)^{-5} - \left(\frac{r}{a}\right)^{-7}\right] \end{aligned} \tag{7}$$

where u, v, w are the velocity component in each dimension, $r = \sqrt{x^2+y^2+z^2}$ is the distance from a point in flow to the center of the sphere, a is the radius of the sphere. Using the direct

forcing IBM, we set the velocity on the sphere surface to zero (no-slip boundary condition). Note the solution above is for unbounded shear flow, thus we impose the Dirichlet velocity boundary conditions on both the upper and bottom walls and choose a larger computation domain to reduce the error by periodic boundaries. After the velocity field reaches its steady state, our numerical results are then compared with the analytical solutions. L_1 and L_2 error norms for different velocity components u, v, w are plotted in Fig.**S4.**(B). It is found that the direct forcing IBM in our model presents second-order accuracy.

Fåhræus-Lindqvist effect: Blood Relative Apparent Viscosity

Fåhræus and Lindqvist (*73*) made the noteworthy observation that the apparent viscosity at shear rates $\geq 100\,\mathrm{s}^{-1}$, determined using Poiseuille's law in a capillary viscometer, exhibited a strong dependence on the capillary tube diameter. This phenomenon is called the Fåhræus-Lindqvist effect. For large tube diameters, a constant viscosity plateau was evident. But within the range of 10 and $1000\mu\mathrm{m}$, the apparent viscosity decreases substantially with decreasing tube size, before increasing sharply for tubes smaller than $10\mu\mathrm{m}$. The increase for very small tubes is readily explained by the relative size of red blood cells, which are approximately $8\mu\mathrm{m}$ in diameter, but the behavior at larger tube diameters is more subtle. In this study, we investigate the behavior of healthy RBCs flowing through tubes of varying radius, ranging from $R = 6\mu m$ to $R = 20\mu m$, as illustrated in Fig.**S5.**(A). Then the computed relative apparent viscosity, as a function of tube diameter, is shown in Fig.**S5.**(B). Fig.**S5.** indicates that our numerical simulation qualitatively predicts the Fåhræus-Lindqvist effect. For comparison, we also show the empirical relation established by Pries et al (*74*) based on *in vitro* blood flow. It is found that our model predicts apparent viscosity in good agreement with the empirical relation at the physiological length scales we study in this work.

Zweifach-Fung Effect: RBC Partitioning at a Bifurcation

The partitioning of blood plasma and cells near a vascular bifurcation is complex. In the microcirculation, when RBCs pass through a bifurcating region of a blood vessel, they exhibit a tendency to preferentially flow into the daughter vessel with the higher flow rate, resulting in fewer cells flowing into the vessel with the lower flow rate. This phenomenon, termed the Zweifach-Fung effect (*75*), plays a crucial role in shaping blood flow distribution within the microvascular network. Here we compare simulation results from our model with experimental observations from previous literature (*76*). The radius of the inlet vessel is taken to be $10\mu m$, in order to be close to the value used in experiments, as shown in Fig.**S6.**. The Zweifach-Fung effect is quantified in Fig.**S6.**(B). The parameter η_Q is defined to characterize partition as the ratio of the volumetric flow rate at one daughter branch to the volumetric flow rate at the parent vessel. Similarly, η_N is defined as the ratio for cell number flow rate. From the results in Fig.**S6.**, it is found that our simulation results are in good agreement with the experimental findings (*76*). Further results on the flow of diseased blood in bifurcations and junctions will be reported elsewhere.

Impact of Membrane Spontaneous Curvature on RBC Dynamics

Although efforts to understand RBC dynamics numerically have spanned the past two decades, the majority of these works have focused on RBC dynamics in cases where the RBC shape is symmetric across the shear plane or where the dimple is centered on the shear plane. Dupont et al. (*77*) demonstrated that an elastic capsule with a prolate spheroid rest shape, whose axis of symmetry is oriented off the shear plane, will exhibit a unique final dynamical motion for all initial orientations. Depending on the capillary number, they observed three final dynamical states: (i) rolling for lower capillary numbers, (ii) wobbling in which the capsule processes around the vorticity axis as the capillary number is increased, and (iii) a swinging-oscillating

motion in which the long axis of the capsule oscillates around the shear plane with decreasing amplitude of oscillation as the capillary number increases, resulting in an in-plane swinging motion at high capillary numbers. Wang et al. (*78*) investigated the off-plane motion of oblate and prolate capsules and concluded that the final dynamical state could depend on the initial inclination angle. A recent study of RBCs in shear flow (*79*) has demonstrated that RBCs first tumble, then roll, transit to a rolling and tumbling stomatocyte, and finally attain polylobed shapes with increasing shear rate when the viscosity contrast between cytosol and blood plasma is large enough. Minetti et al. (*80*) give an exhaustive description of the dynamics under a shear flow of a large number of RBCs in a dilute regime is proposed. They identify which of the characteristic parameters of motion and of the transition thresholds depend on flow stress only or also on suspending fluid viscosity.

Similarly, Cordosco and Bagchi (*81*) studied the off-plane motion of oblate, prolate, and biconcave capsules. Unlike Dupont et al. (*77*) and Wang et al. (*78*), they included membrane bending stiffness in their formulation and considered a spatially uniform spontaneous curvature in the case of biconcave capsules. They found that rolling was the dominant mode in the physiologically relevant viscosity ratio case (i.e., 5), tank-treading or wobbling mode at $\lambda < 1$, and an intermittent regime at low capillary numbers and low viscosity ratios, where the dynamics are dependent on the initial orientation. It is noteworthy that Bitbol (*82*) and Dupire et al. (*77*) experimentally observed rolling dynamics in a dextran solution where the viscosity ratio was less than unity. The discrepancy between simulation and experiment may result from the use of a spatially uniform spontaneous curvature that corresponds to a biconcave shape. It is important to note that to model the RBC membrane correctly, an assumption of the spontaneous shape has to be made, and finding the appropriate shape has been a challenge for both theoreticians and experimentalists. Sinha et al. (*47*) investigate the cell dynamics' dependence on the membrane's spontaneous curvature. They found that an oblate spheroidal spontaneous curvature maintains

the dimple of the RBC during tank-treading dynamics and exhibits off-shear-plane, tumbling consistent with the experimental observations of Dupire et al. (*45*). For a complex structure such as an RBC membrane, it is possible that the natural shape for shear elasticity may differ from that for bending elasticity so the overall natural shape of an element results from the balance of bending and shear forces.

There have been endeavors to comprehend the impact of spontaneous shape on the ultimate dynamics of RBCs. Peng et al. (*83*) conducted a study on the influence of non-biconcave spontaneous shape on RBC dynamics and concluded that in order for an RBC to maintain its biconcave shape during tank-treading, as noted by Dupire et al. (*45*), the spontaneous curvature must be non-biconcave. In instances where a biconcave spontaneous curvature was employed, tank-treading could not be achieved without significantly perturbing the initial shape. Additionally, Cordosco et al. (*84*) explored non-biconcave spontaneous shapes and ascertained that the spontaneous shape has a significant impact on cell dynamics, depending on the viscosity ratio. They observed that the dimple in the RBC remained intact for both biconcave and oblate spontaneous shapes. However, it should be noted that in both works, Peng et al. (*83*) and Cordasco et al. (*84*), non-biconcave spontaneous curvatures were investigated under the imposition of spatially uniform spontaneous curvature, denoted as c_0. It is worth highlighting that RBC membranes differ from model lipid bilayers in that they possess embedded proteins with an underlying spectrin cytoskeleton and an asymmetric bilayer leaflet composition, all of which modify c_0, with proteins in particular, having been demonstrated to preferentially bind via curvature-sensing mechanisms. Hence, it can be argued that c_0 would be spatially inhomogeneous. Recently, using two different simulation techniques, Mauer et al. (*85*) construct a state diagram of RBC shapes and dynamics in shear flow as a function of shear rate and viscosity contrast (*45, 86*). Their studies suggest that a nearly spherical stress-free shape best reproduces experimental results for the tumbling-to-tank-treading transition at low viscos-

ity contrasts. Reichel et al. (*87*) combined simulation and experimental investigation of RBC shapes and dynamics in microchannels to provide a consistent RBC state diagram and illustrate the complexity of RBC behavior in the microflow. The RBC model employs a stress-free shape of the elastic spring network, corresponding to a spheroidal shape with a reduced volume of 0.96. Their simulation results agree well with experimental observations, allow the characterization of RBC variability in shear elasticity, and permit us to make a significant step toward quantitative measurements of RBC mechanical properties.

In our work, to investigate the effects of membrane curvature on the RBC dynamics, two types of homogeneous normal RBCs suspension are simulated: one with spontaneous bending curvature being biconcave discoid, while another being oblate spheroid. Various vascular geometries are considered, including a slit (Fig.**S7.**), a straight cylindrical tube (Fig. **S8.**), and a curved (serpentine) channel (Fig. **S9.**). The simulation snapshots tell that RBC rest shape has a nontrivial impact on its near-wall dynamics: RBCs with biconcave discoid rest shape tend to ”rolling”, while RBCs with oblate spheroid rest shape perform ”tank-treading”, consistent with the findings in a prior numerical investigation by Sinha et al. (*47*). In spite of the change in orientational dynamics when the spontaneous shape is changed, no substantial change is observed in the number density distribution for normal RBCs.

Simulation snapshots of Cell Distributions in Straight Cylindrical Tube

The simulation snapshots showing the segregation phenomenon between normal and aberrant cells in the straight cylindrical tube are given in Fig. **S10.**.

RBC-induced Local Wall Shear Stress Fluctuation on Curved Channel Surface

Cross-sectional and center-plane normalized cell number density distribution of iron deficiency RBCs, sphero-echinocytes, and spherocytes over the curved channel in each corresponding

binary suspension of normal RBCs with aberrant RBCs are shown in Fig.**S11.**. Aberrant RBCs margination provokes local wall shear stress fluctuation on curved channel surfaces are shown in Fig. **S12.**, **S13.**, and **S14.**.

Movies

Movie S1.

SCD RBC suspension in Straight Cylindrical Tube

Movie S2.

IDA RBC suspension in Straight Cylindrical Tube

Movie S3.

COVID-19 RBC suspension in Straight Cylindrical Tube

Movie S4.

Spherocytosis RBC suspension in Straight Cylindrical Tube

Movie S5.

SCD RBC suspension in Curved Channel

Movie S6.

IDA RBC suspension in Curved Channel

Movie S7.

COVID-19 RBC suspension in Curved Channel

Movie S8.

Spherocytosis RBC suspension in Curved Channel

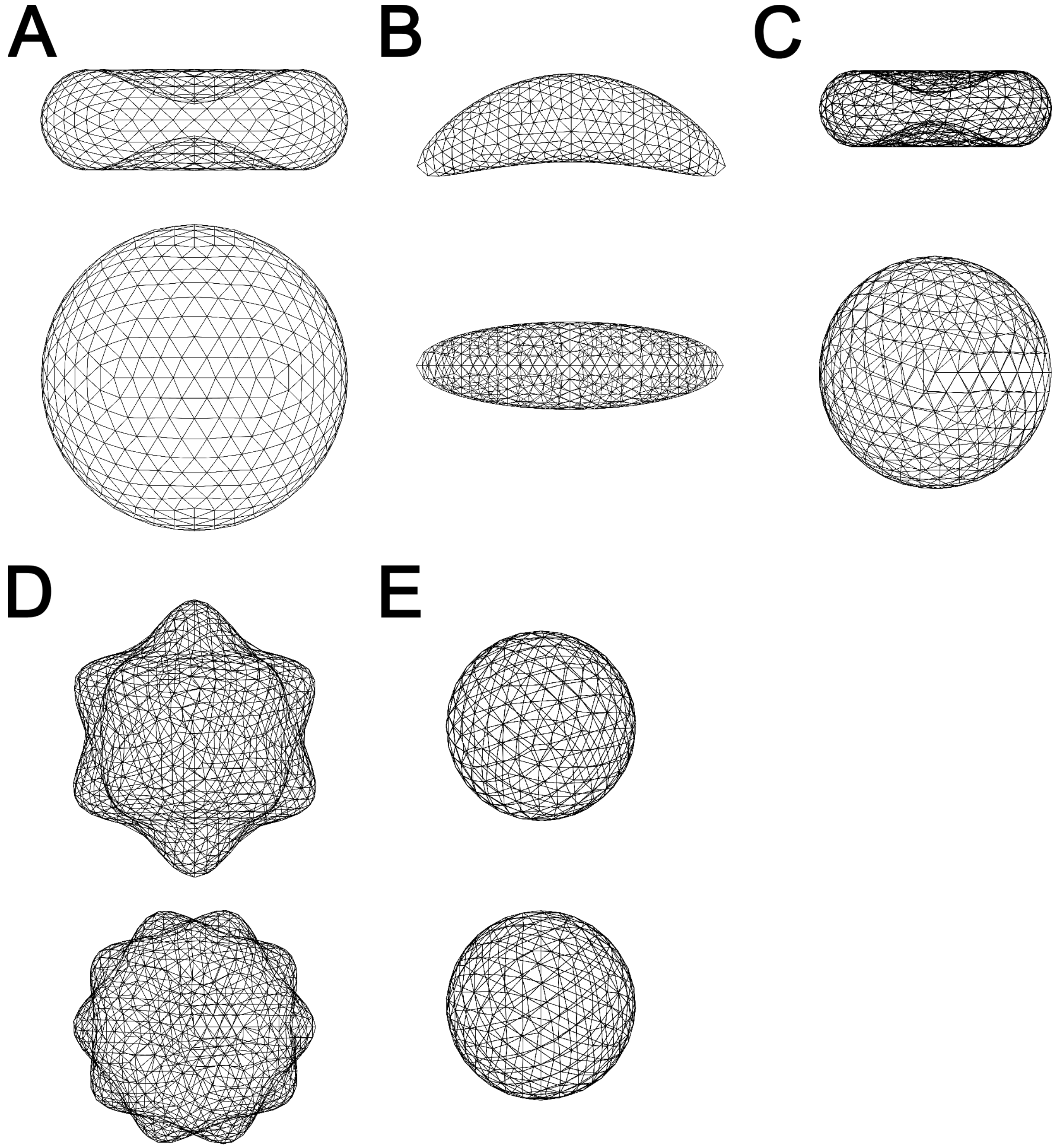

Fig. S2. The surface mesh for capsules of (A) biconcave-discoid normal RBC, (B) sickle RBC, (C) iron deficiency RBC, (D) sphere-echinocyte, and (E) spherocyte. Note the size of each cell species in this figure is corresponding to the cell used in our simulation. The radius of normal RBC is $4\mu m$; aberrant RBCs are smaller than normal RBCs.

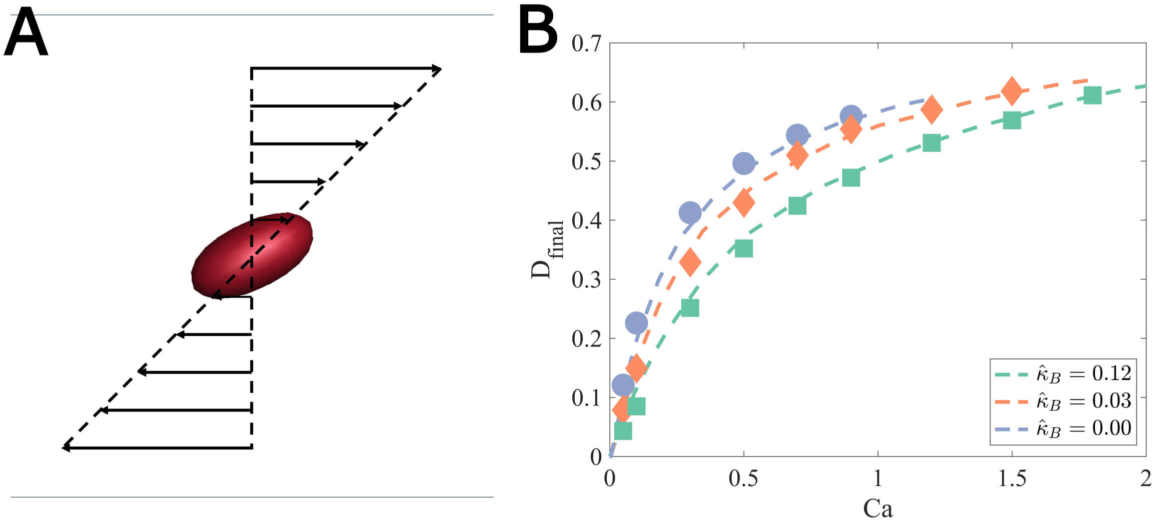

Fig. S3. (A) Schematic diagram of an elastic spherical capsule in simple shear flow. Velocity Dirichlet boundary conditions are imposed on both the top and bottom plates to create a simple shear flow. Because of the existence of a constant shear rate, the sphere capsule is stretched and then deformed into an ellipsoid. (B) Steady-state Taylor deformation parameter for a spherical capsule as a function of Ca. Dash lines are results from previous literature (*72*), and symbols are our simulation results.

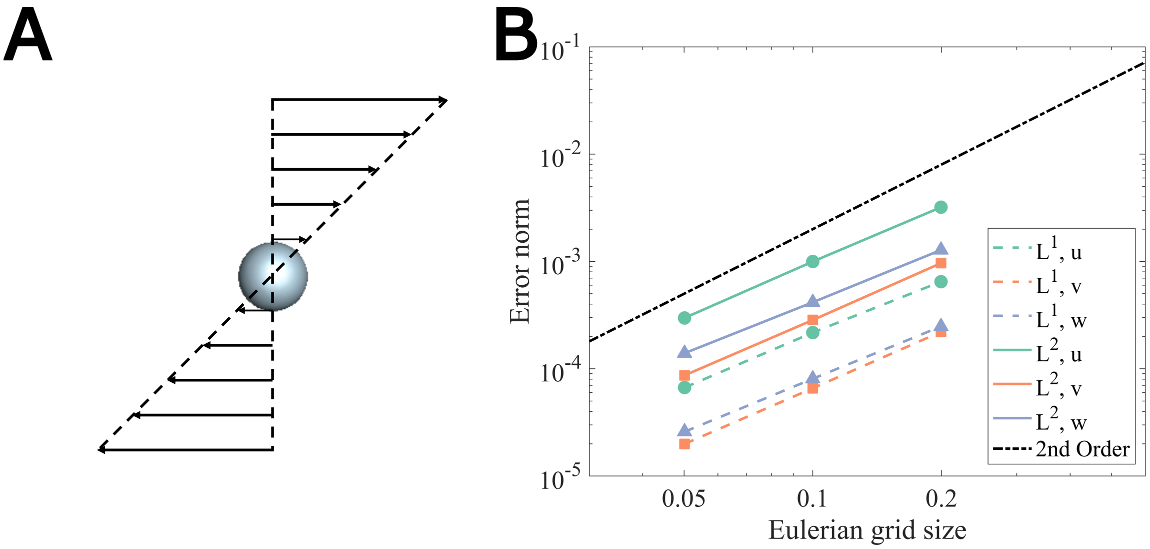

Fig. S4. (A) Schematic of simulating a rigid stationary sphere subjected to a linear shear flow. The velocity field on the top and bottom wall is set to be the analytical solution (Dirichlet boundary condition). Periodic boundary conditions are imposed along x and y direction. A rigid sphere is placed at the center of the box. The velocity on the sphere surface is set to zero. (B) Error norm vs. mesh size for stationary rigid sphere in a linear shear flow

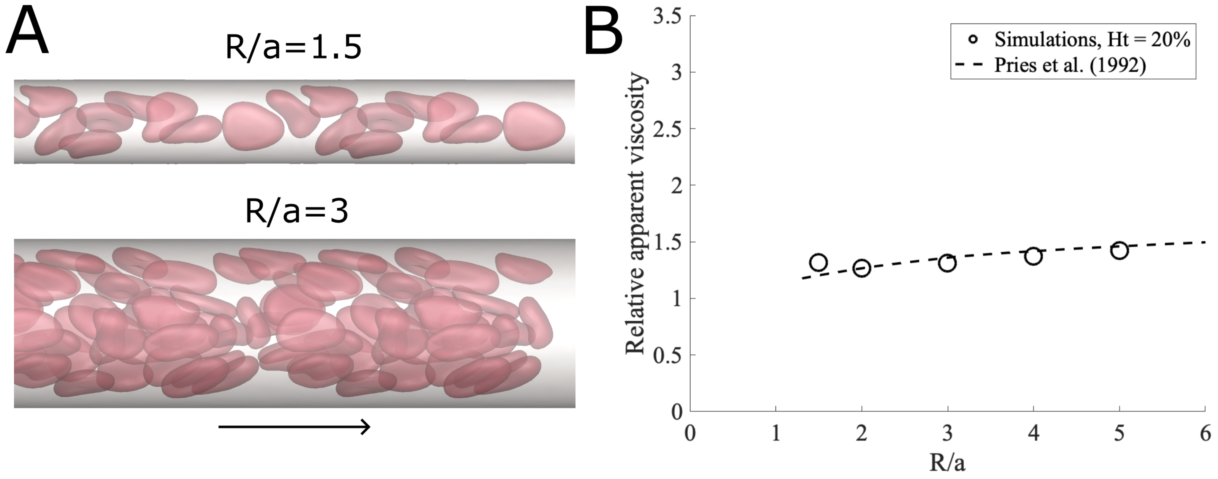

Fig. S5. (A) Simulation snapshots of healthy RBCs with radius $a = 4\mu m$ flowing through tubes of various radius R. Hematocrit is set to be 20%. The arrow indicates the direction of flow. (B) The relative apparent viscosity as a function of tube radius R and comparison with experimental relation in (*74*).

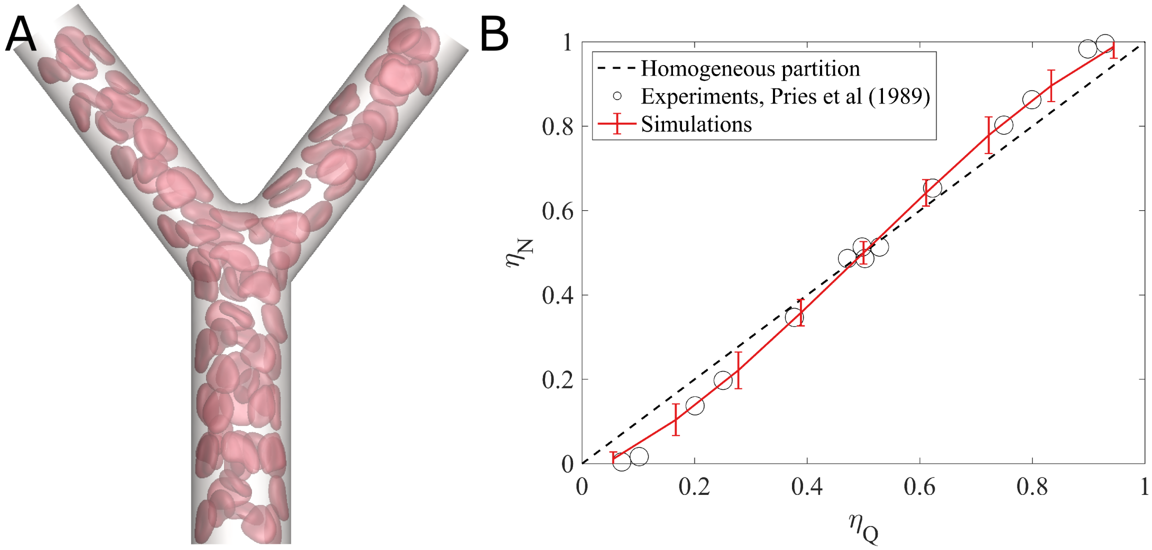

Fig. S6. (A) Simulation snapshots of healthy RBCs partitioning near a bifurcation. The radius of the inlet vessel is $10\mu m$. (B) Comparison of the Zweifach-Fung effect in our simulation with experimental results from past literature (*76*).

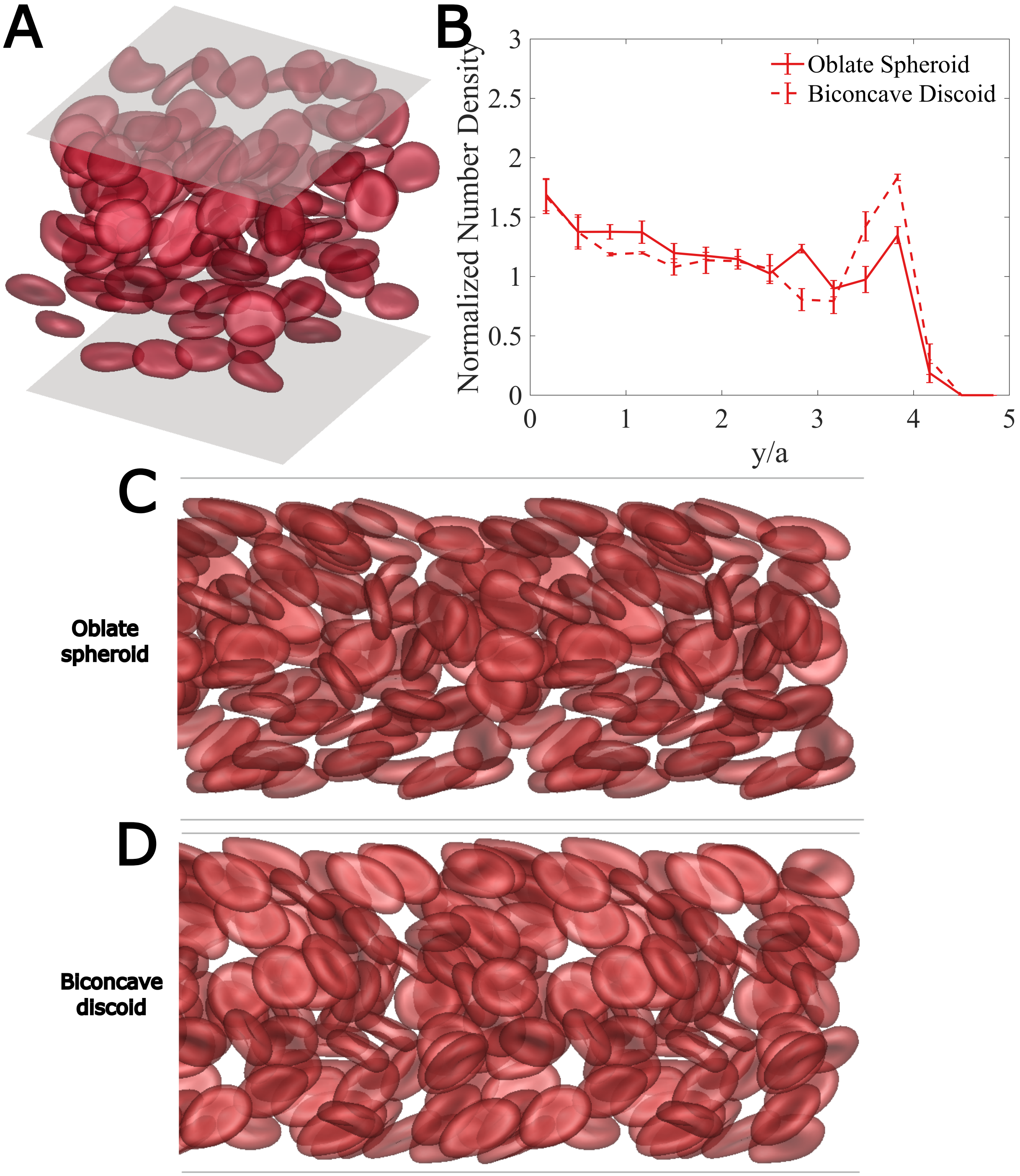

Fig. S7. (Top) (A) Simulation diagram of homogeneous normal RBC suspension between the slit under pressure-driven flow. (Re_p = 0.1, Hematocrit = 0.15)(B) Steady-state wall-normal direction cell number density profile. Note here $y/a = 0$ denotes the slit center and $y/a = 5$ close to the wall. (Bottom) The side view of RBC suspension with bending spontaneous curvature being (C) oblate spheroid and (D) biconcave discoid.

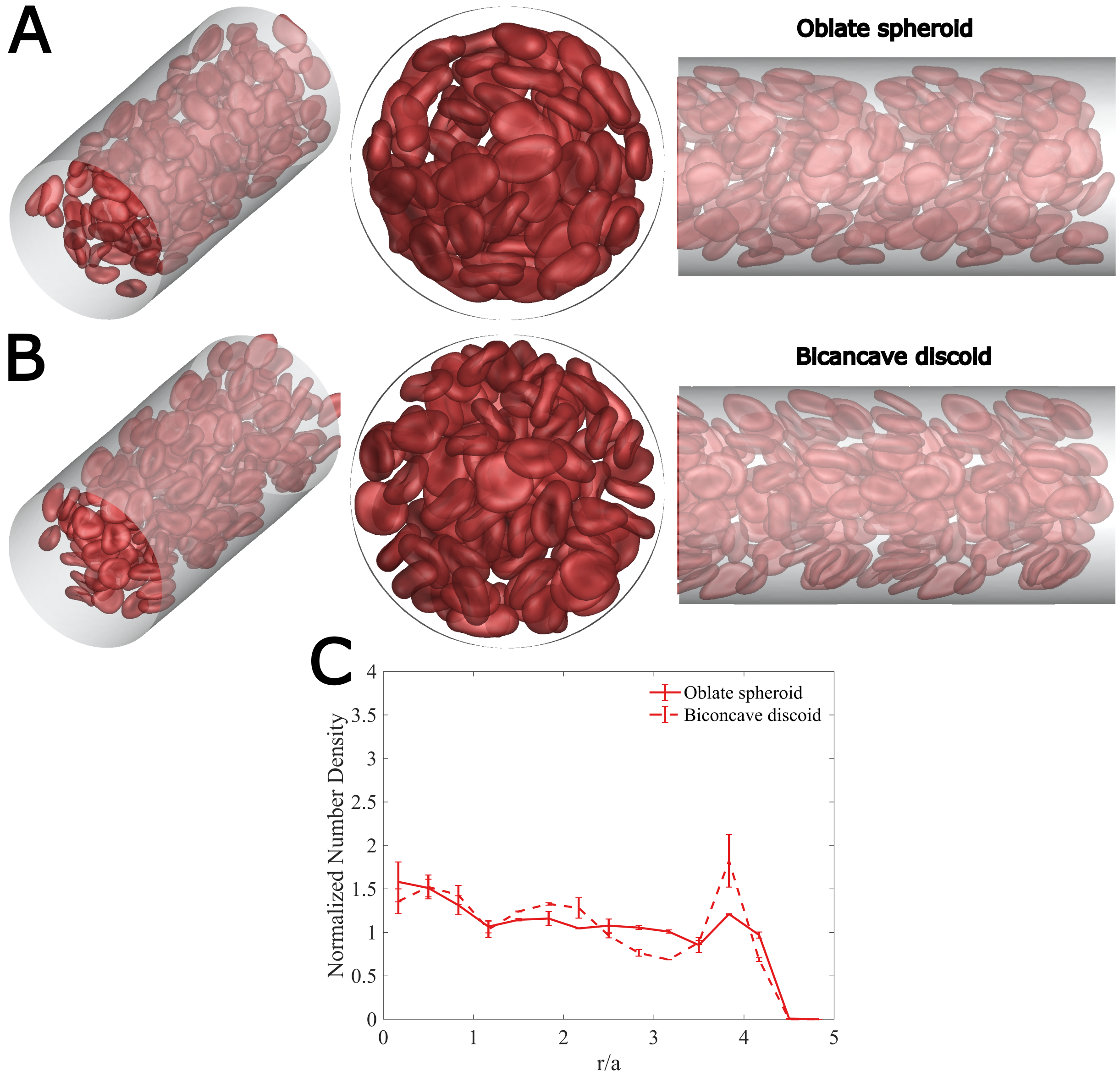

Fig. S8. (Top)Simulation snapshots for homogeneous normal RBC suspension within the straight cylindrical tube with spontaneous bending curvature being (A) oblate spheroid and (B) biconcave discoid. (Re_p=0.1, Hematocrit = 0.20). (Bottom) (C) Steady-state radial cell number density profile for two types of RBC suspensions.

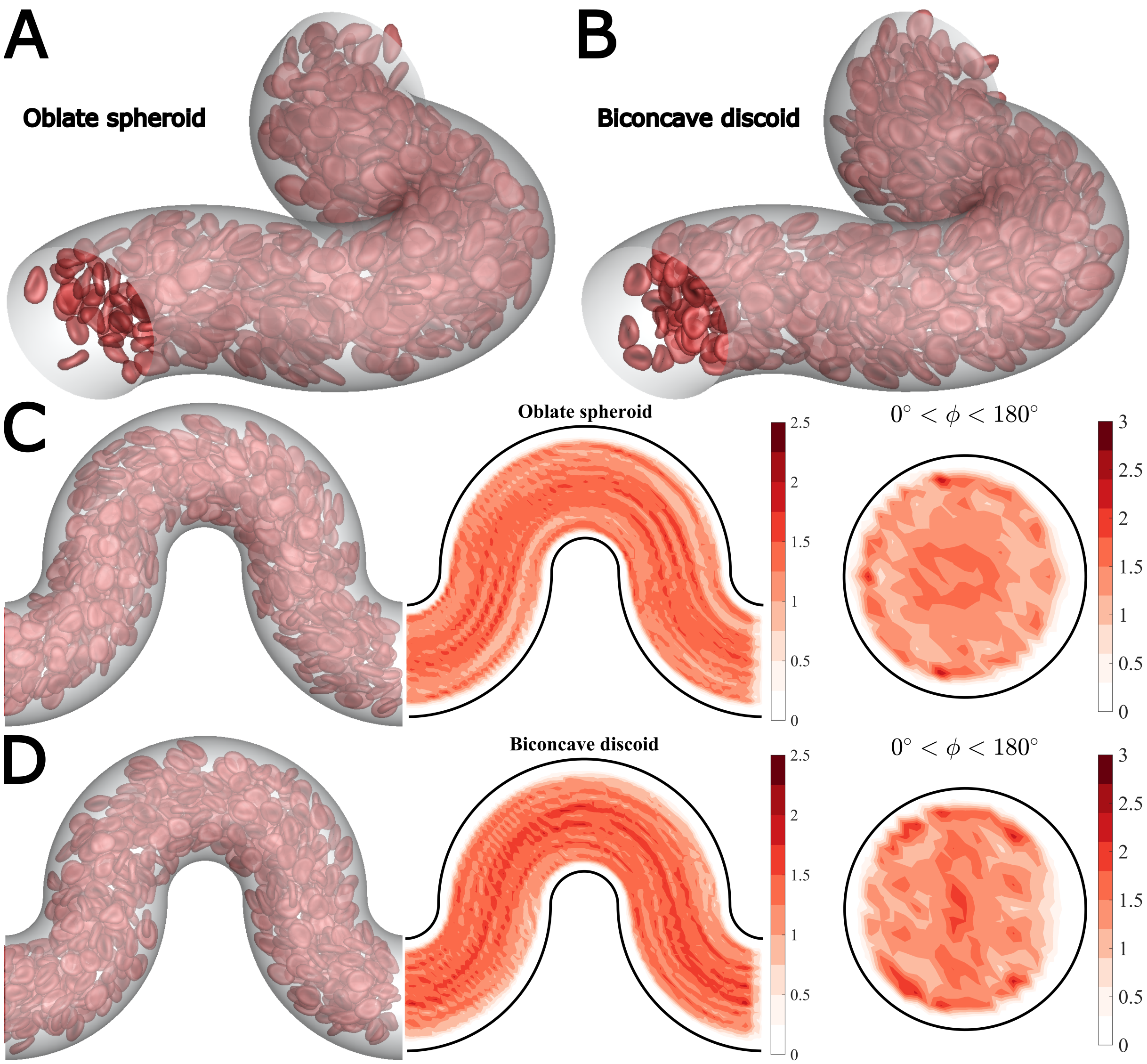

Fig. S9. (Top) Simulation snapshots for normal RBC suspension in the curved serpentine channel with spontaneous curvature being (A) oblate spheroid and (B) biconcave discoid. (Bottom) Center-plane and cross-sectional cell number density distribution for RBC suspension with spontaneous curvature being (C) oblate spheroid and (D) biconcave discoid.

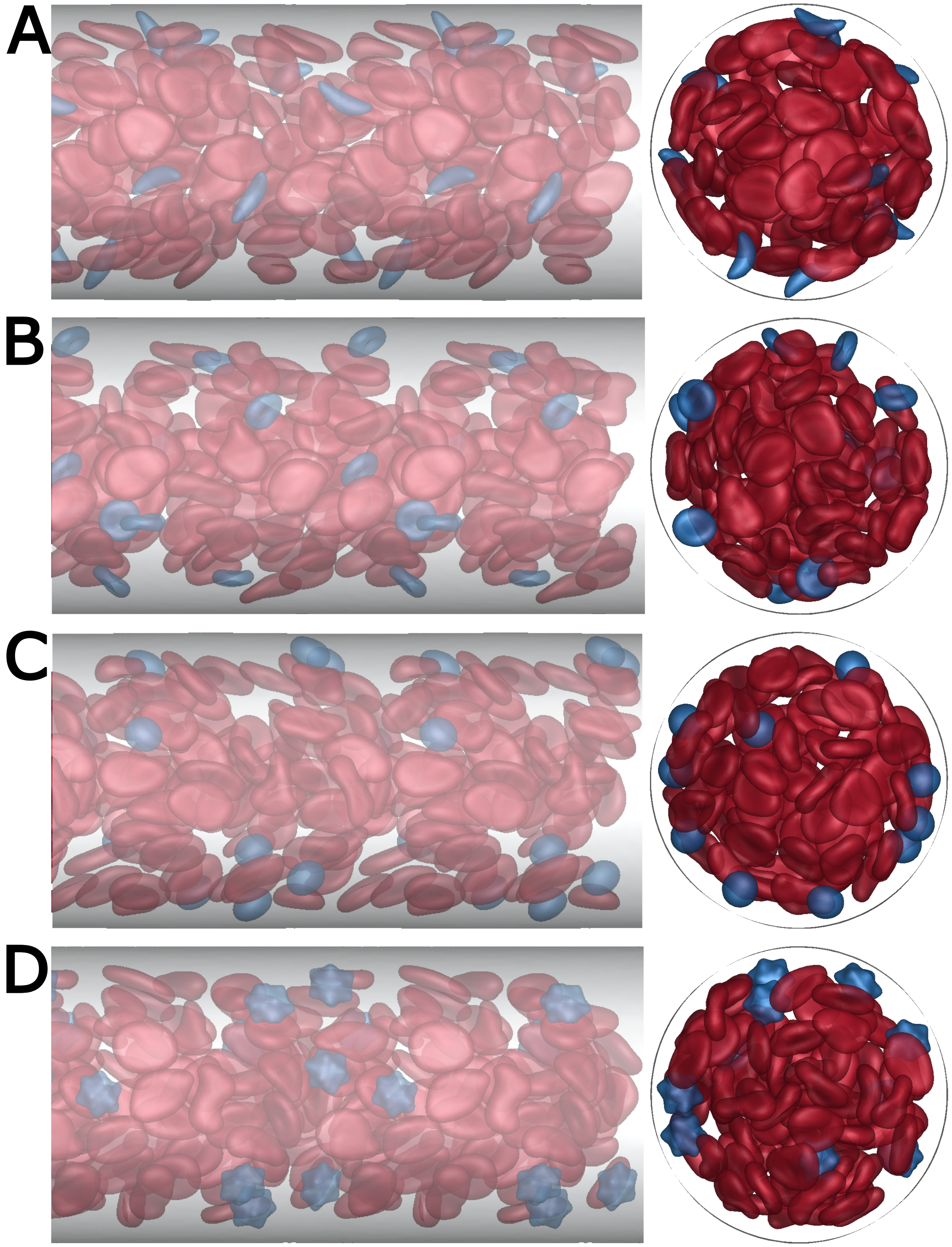

Fig. S10. Simulation snapshots (left: side view; right: top view) for (A) SCD, (B) IDA, (C) spherocytosis, and, (D) COVID-19 RBC suspension in the cylindrical straight channel.

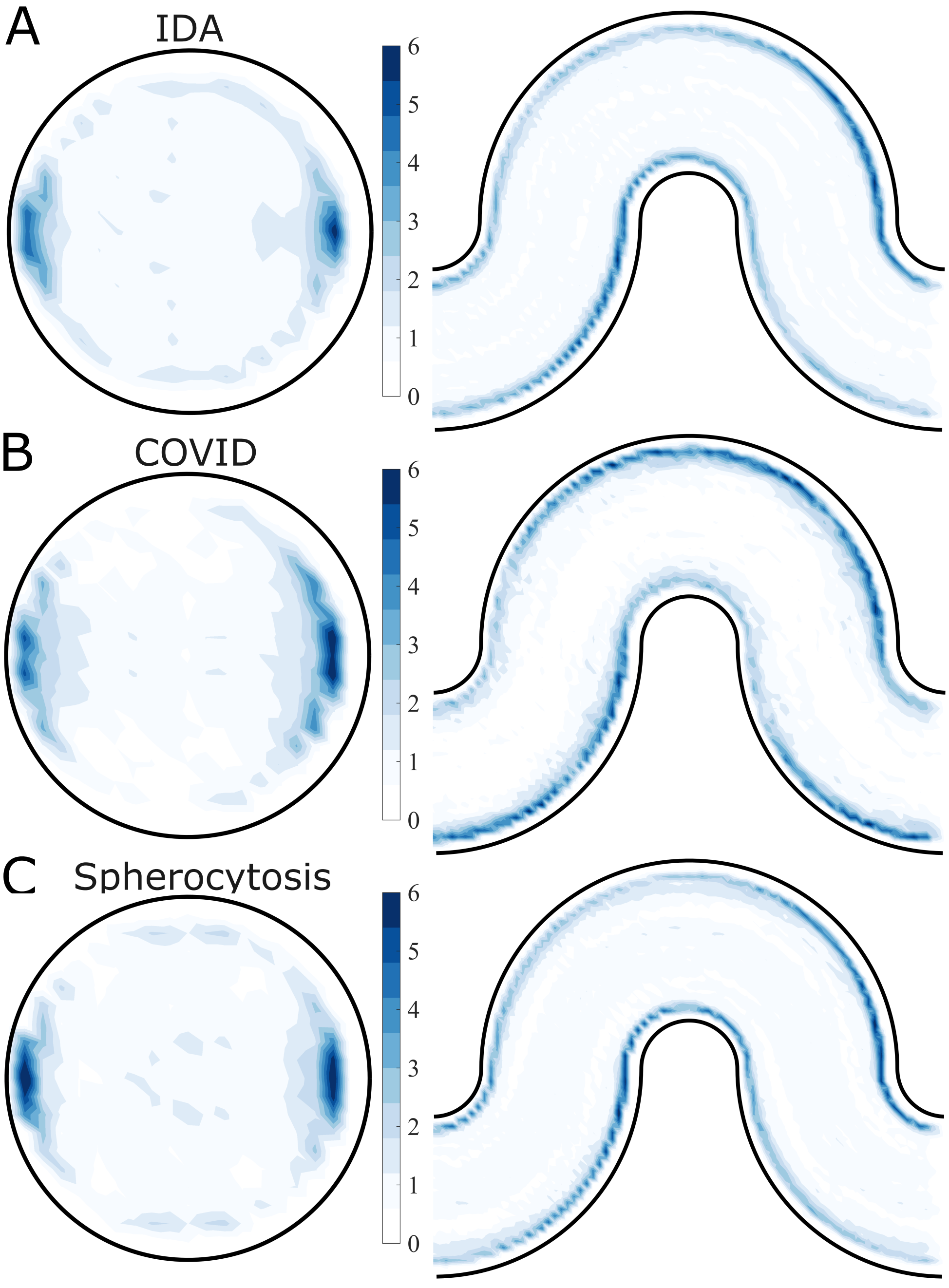

Fig. S11. Distributions of aberrant cells within a curved blood vessel. Cross-sectional and center-plane normalized cell number density distribution of (A) iron deficiency RBCs, (B) sphero-echinocytes, and (C) spherocytes over the entire curved channel ($0° < \theta < 180°$) in each corresponding binary suspensions of normal RBCs with aberrant RBCs.

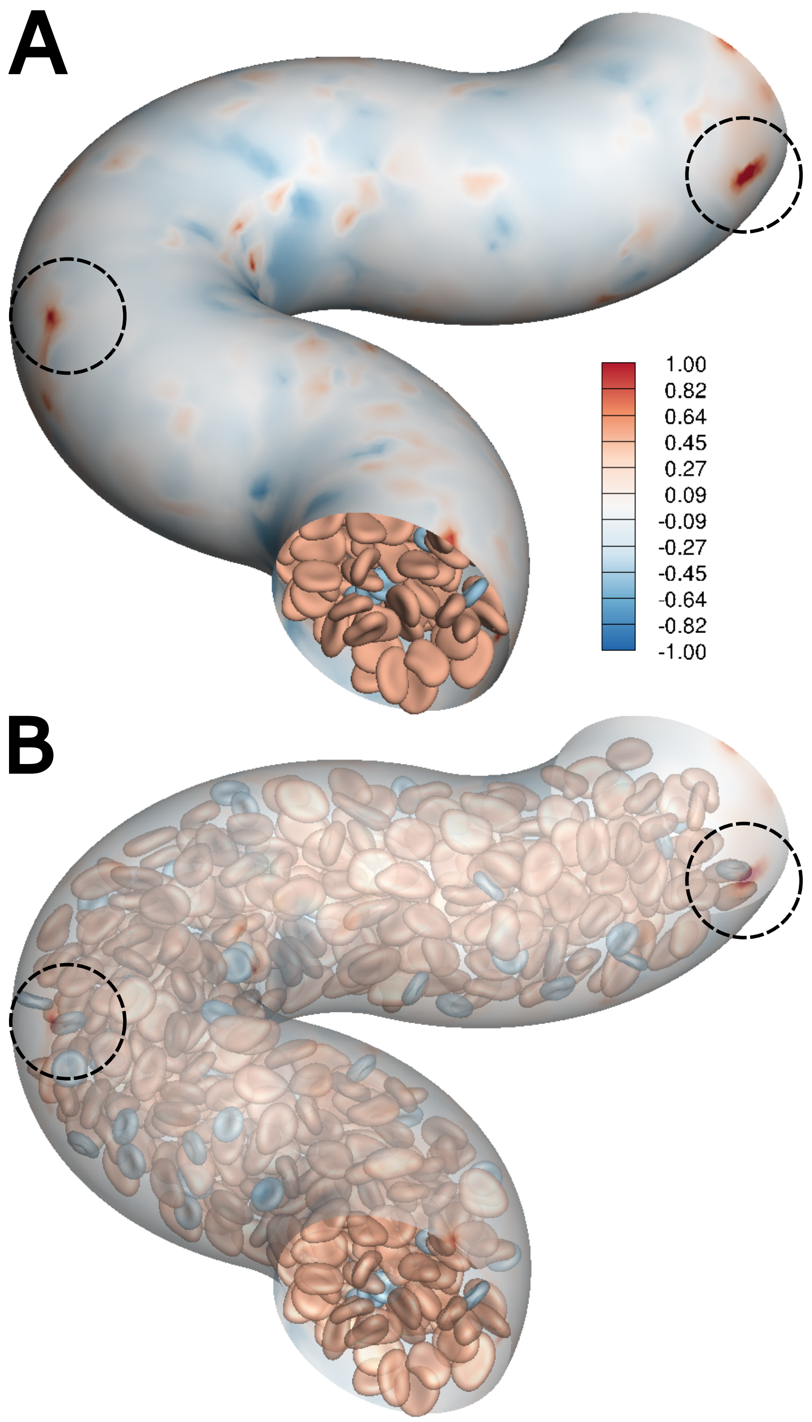

Fig. S12. (A) Simulation snapshots and (B) corresponding transparent views of additional wall shear stress $\hat{\tau}_w$ induced by the presence of the cells in suspensions of normal RBCs with iron deficiency RBCs within the serpentine channel. The color on the serpentine vascular surface denotes the RBC-induced wall shear stress strength $\hat{\tau}_w$.

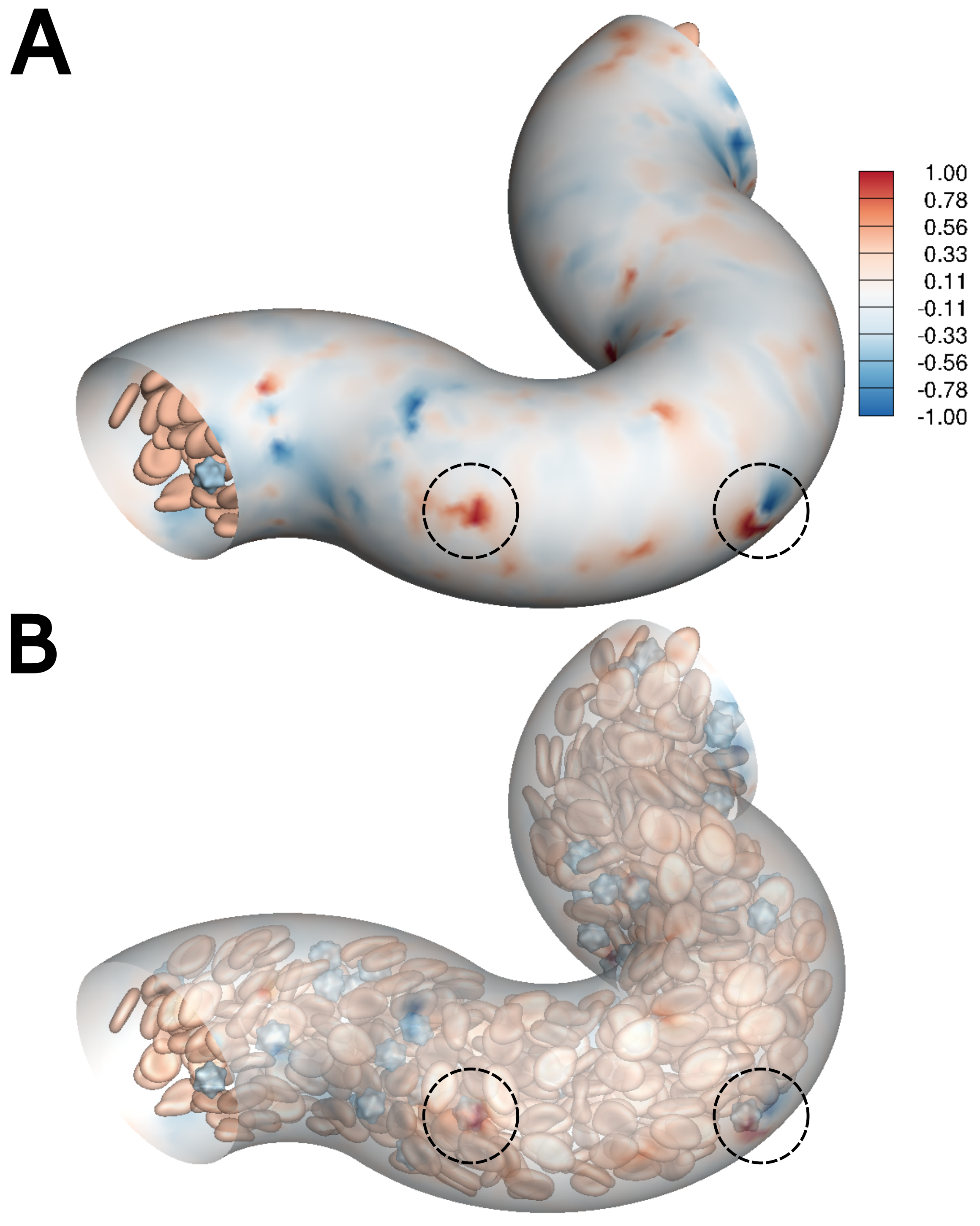

Fig. S13. (A) Simulation snapshots and (B) corresponding transparent views of additional wall shear stress $\hat{\tau}_w$ induced by the presence of the cells in suspensions of normal RBCs with sphero-echinocytes within the serpentine channel. The color on the serpentine vascular surface denotes the RBC-induced wall shear stress strength $\hat{\tau}_w$.

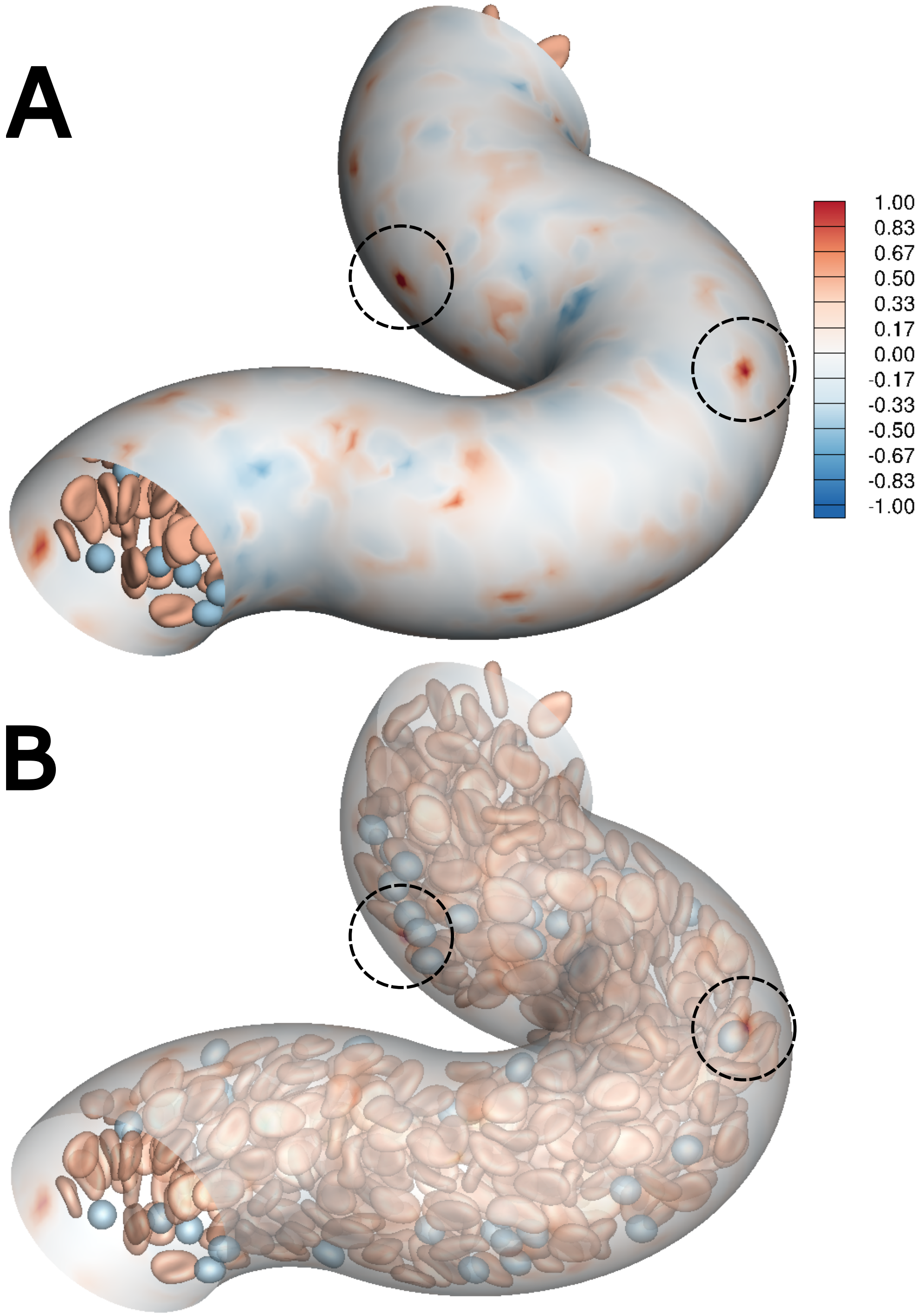

Fig. S14. (A) Simulation snapshots and (B) corresponding transparent views of additional wall shear stress $\hat{\tau}_w$ induced by the presence of the cells in suspensions of normal RBCs with spherocytes within the serpentine channel. The color on the serpentine vascular surface denotes the RBC-induced wall shear stress strength $\hat{\tau}_w$.

REFERENCES AND NOTES

1. D. C. Rees, T. N. Williams, M. T. Gladwin, Sickle-cell disease. *Lancet* **376**, 2018–2031 (2010).

2. S. M. Recktenwald, G. Simionato, M. G. Lopes, F. Gamboni, M. Dzieciatkowska, P. Meybohm, K. Zacharowski, A. von Knethen, C. Wagner, L. Kaestner, A. D'Alessandro, S. Quint, Cross-talk between red blood cells and plasma influences blood flow and omics phenotypes in severe COVID-19. *eLife* **11**, e81316 (2022).

3. S. Druzak, E. Iffrig, B. R. Roberts, T. Zhang, K. S. Fibben, Y. Sakurai, H. P. Verkerke, C. A. Rostad, A. Chahroudi, F. Schneider, A. K. H. Wong, A. M. Roberts, J. D. Chandler, S. O. Kim, M. Mosunjac, M. Mosunjac, R. Geller, I. Albizua, S. R. Stowell, C. M. Arthur, E. J. Anderson, A. A. Ivanova, J. Ahn, X. Liu, K. Maner-Smith, T. Bowen, M. Paiardini, S. E. Bosinger, J. D. Roback, D. A. Kulpa, G. Silvestri, W. A. Lam, E. A. Ortlund, C. L. Maier, Multiplatform analyses reveal distinct drivers of systemic pathogenesis in adult versus pediatric severe acute COVID-19. *Nat. Commun.* **14**, 1638 (2023).

4. J. M. O'Brien Jr, N. A. Ali, S. K. Aberegg, E. Abraham, Sepsis. *Am. J. Med.* **120**, 1012–1022 (2007).

5. S. Von Haehling, E. A. Jankowska, D. J. Van Veldhuisen, P. Ponikowski, S. D. Anker, Iron deficiency and cardiovascular disease. *Nat. Rev. Cardiol.* **12**, 659–669 (2015).

6. S. Perrotta, P. G. Gallagher, N. Mohandas, Hereditary spherocytosis. *Lancet* **372**, 1411–1426 (2008).

7. C. Hahn, M. A. Schwartz, Mechanotransduction in vascular physiology and atherogenesis. *Nat. Rev. Mol. Cell Biol.* **10**, 53–62 (2009).

8. B. K. Walther, N. K. R. Pandian, K. A. Gold, E. S. Kiliç, V. Sama, J. Gu, A. K. Gaharwar, A. Guiseppi-Elie, J. P. Cooke, A. Jain, Mechanotransduction-on-chip: Vessel-chip model of endothelial YAP mechanobiology reveals matrix stiffness impedes shear response. *Lab Chip* **21**, 1738–1751 (2021).

9. K.-C. Wang, Y.-T. Yeh, P. Nguyen, E. Limqueco, J. Lopez, S. Thorossian, K.-L. Guan, Y.-S. J. Li, S. Chien, Flow-dependent YAP/TAZ activities regulate endothelial phenotypes and atherosclerosis. *Proc. Natl. Acad. Sci. U.S.A.* **113**, 11525–11530 (2016).

10. M. He, M. Martin, T. Marin, Z. Chen, B. Gongol, Endothelial mechanobiology. *APL Bioeng.* **4**, 010904 (2020).

11. T. Panciera, L. Azzolin, M. Cordenonsi, S. Piccolo, Mechanobiology of YAP and TAZ in physiology and disease. *Nat. Rev. Mol. Cell Biol.* **18**, 758–770 (2017).

12. X. Zhou, W. Xu, Y. Xu, Z. Qian, Iron supplementation improves cardiovascular outcomes in patients with heart failure. *Am. J. Med.* **132**, 955–963 (2019).

13. C. Souilhol, J. Serbanovic-Canic, M. Fragiadaki, T. J. Chico, V. Ridger, H. Roddie, P. C. Evans, Endothelial responses to shear stress in atherosclerosis: A novel role for developmental genes. *Nat. Rev. Cardiol.* **17**, 52–63 (2020).

14. E. Tzima, M. Irani-Tehrani, W. B. Kiosses, E. Dejana, D. A. Schultz, B. Engelhardt, G. Cao, H. DeLisser, M. A. Schwartz, A mechanosensory complex that mediates the endothelial cell response to fluid shear stress. *Nature* **437**, 426–431 (2005).

15. R. Mannino, D. R. Myers, B. Ahn, H. Gole, Y. Wang, A. Lin, R. Guldberg, D. Giddens, L. Timmins, W. A. Lam, Vascular geometry and flow profile mediate pathological cell-cell interactions in sickle cell disease as measured with "do-it-yourself" "endothelial-ized" microfluidics. *Blood* **124**, 454 (2014).

16. Y. Wang, R. G. Mannino, D. R. Myers, W. Li, C. H. Joiner, W. A. Lam, Vessel geometry interacts with red blood cell stiffness to promote endothelial dysfunction in sickle cell disease. *Blood* **126**, 965 (2015).

17. J. F. Bertles, P. F. Milner, Irreversibly sickled erythrocytes: A consequence of the heterogeneous distribution of hemoglobin types in sickle-cell anemia. *J. Clin. Investig.* **47**, 1731–1741 (1968).

18. C. Caruso, M. E. Fay, X. Cheng, A. Y. Liu, S. I. Park, T. A. Sulchek, M. D. Graham, W. A. Lam, Pathologic mechanobiological interactions between red blood cells and endothelial cells directly induce vasculopathy in iron deficiency anemia. *iScience* **25**, 104606 (2022).

19. J. C. Firrell, H. H. Lipowsky, Leukocyte margination and deformation in mesenteric venules of rat. *Am. J. Physiol.* **256**, H1667–H1674 (1989).

20. G. J. Tangelder, H. C. Teirlinck, D. W. Slaaf, R. S. Reneman, Distribution of blood platelets flowing in arterioles. *Am. J. Physiol.* **248**, H318–H323 (1985).

21. J. J. Bishop, A. S. Popel, M. Intaglietta, P. C. Johnson, Effect of aggregation and shear rate on the dispersion of red blood cells flowing in venules. *Am. J. Physiol. Heart Circ. Physiol.* **283**, H1985–H1996 (2002).

22. V. Clavería, P. Connes, L. Lanotte, C. Renoux, P. Joly, R. Fort, A. Gauthier, C. Wagner, M. Abkarian, In vitro red blood cell segregation in sickle cell anemia. *Front. Phys.* **9**, 712 (2021).

23. E. Evans, N. Mohandas, A. Leung, Static and dynamic rigidities of normal and sickle erythrocytes. Major influence of cell hemoglobin concentration. *J. Clin. Invest.* **73**, 477–488 (1984).

24. A. Kumar, M. D. Graham, Segregation by membrane rigidity in flowing binary suspensions of elastic capsules. *Phys. Rev. E Stat. Nonlin. Soft Matter. Phys.* **84**, 066316 (2011).

25. A. Kumar, R. G. H. Rivera, M. D. Graham, Flow-induced segregation in confined multicomponent suspensions: Effects of particle size and rigidity. *J. Fluid Mech.* **738**, 423–462 (2014).

26. K. Sinha, M. D. Graham, Shape-mediated margination and demargination in flowing multicomponent suspensions of deformable capsules. *Soft Matter* **12**, 1683–1700 (2016).

27. Q. M. Qi, E. S. G. Shaqfeh, Theory to predict particle migration and margination in the pressure-driven channel flow of blood. *Phys. Rev. Fluids* **2**, 093102 (2017).

28. K. Mueller, D. A. Fedosov, G. Gompper, Understanding particle margination in blood flow—A step toward optimized drug delivery systems. *Med. Eng. Phys.* **38**, 2–10 (2016).

29. A. Kumar, M. D. Graham, Mechanism of margination in confined flows of blood and other multicomponent suspensions. *Phys. Rev. Lett.* **109**, 108102 (2012).

30. V. Narsimhan, H. Zhao, E. S. G. Shaqfeh, Coarse-grained theory to predict the concentration distribution of red blood cells in wall-bounded Couette flow at zero Reynolds number. *Phys. Fluids* **25**, 061901 (2013).

31. R. G. H. Rivera, K. Sinha, M. D. Graham, Margination regimes and drainage transition in confined multicomponent suspensions. *Phys. Rev. Lett.* **114**, 188101 (2015).

32. R. G. H. Rivera, X. Zhang, M. D. Graham, Mechanistic theory of margination and flow-induced segregation in confined multicomponent suspensions: Simple shear and poiseuille flows. *Phys. Rev. Fluids* **1**, 060501 (2016).

33. C. Collins, L. D. Osborne, C. Guilluy, Z. Chen, E. T. O'Brien III, J. S. Reader, K. Burridge, R. Superfine, E. Tzima, Haemodynamic and extracellular matrix cues regulate the mechanical phenotype and stiffness of aortic endothelial cells. *Nat. Commun.* **5**, 3984 (2014).

34. J. Eyckmans, T. Boudou, X. Yu, C. S. Chen, A hitchhiker's guide to mechanobiology. *Dev. Cell* **21**, 35–47 (2011).

35. D. Harrison, J. Widder, I. Grumbach, W. Chen, M. Weber, C. Searles, Endothelial mechanotransduction, nitric oxide and vascular inflammation. *J. Intern. Med.* **259**, 351–363 (2006).

36. D. E. Ingber, From mechanobiology to developmentally inspired engineering. *Philos. Trans. R. Soc. Lond. B Biol. Sci.* **373**, 20170323 (2018).

37. D. Ingber, Mechanobiology and diseases of mechanotransduction. *Ann. Med.* **35**, 564–577 (2003).

38. J.-I. Abe, B. C. Berk, Novel mechanisms of endothelial mechanotransduction. *Arterioscler. Thromb. Vasc. Biol.* **34**, 2378–2386 (2014).

39. C. Caruso, X. Zhang, Y. Sakurai, W. Li, M. E. Fay, M. A. Carden, D. R. Myers, R. G. Mannino, C. H. Joiner, M. D. Graham, W. A. Lam, Stiff erythrocyte subpopulations biomechanically induce endothelial inflammation in sickle cell disease. *Blood* **134**, 3560 (2019).

40. X. Zhang, C. Caruso, W. A. Lam, M. D. Graham, Flow-induced segregation and dynamics of red blood cells in sickle cell disease. *Phys. Rev. Fluids* **5**, 053101 (2020).

41. B. Klitzman, B. R. Duling, Microvascular hematocrit and red cell flow in resting and contracting striated muscle. *Am. J. Physiol. Heart Circ. Physiol.* **237**, H481–H490 (1979).

42. I. H. Sarelius, B. R. Duling, Direct measurement of microvessel hematocrit, red cell flux, velocity, and transit time. *Am. J. Physiol.* **243**, H1018–H1026 (1982).

43. H. Byun, T. R. Hillman, J. M. Higgins, M. Diez-Silva, Z. Peng, M. Dao, R. R. Dasari, S. Suresh, Y. Park, Optical measurement of biomechanical properties of individual erythrocytes from a sickle cell patient. *Acta Biomater.* **8**, 4130–4138 (2012).

44. N. Mohandas, M. R. Clark, M. S. Jacobs, S. B. Shohet, Analysis of factors regulating erythrocyte deformability. *J. Clin. Invest.* **66**, 563–573 (1980).

45. J. Dupire, M. Socol, A. Viallat, Full dynamics of a red blood cell in shear flow. *Proc. Natl. Acad. Sci. U.S.A.* **109**, 20808–20813 (2012).

46. T. M. Fischer, Shape memory of human red blood cells. *Biophys. J.* **86**, 3304–3313 (2004).

47. K. Sinha, M. D. Graham, Dynamics of a single red blood cell in simple shear flow. *Phys. Rev. E Stat. Nonlin. Soft Matter. Phys.* **92**, 042710 (2015).

48. B. Czaja, J. de Bouter, M. Heisler, G. Závodszky, S. Karst, M. Sarunic, D. Maberley, A. Hoekstra, The effect of stiffened diabetic red blood cells on wall shear stress in a reconstructed 3D microaneurysm. *Comput. Methods Biomech. Biomed. Engin.* **25**, 1691–1709 (2022).

49. S. Ebrahimi, P. Bagchi, A computational study of red blood cell deformability effect on hemodynamic alteration in capillary vessel networks. *Sci. Rep.* **12**, 4304 (2022).

50. C. Bächer, A. Kihm, L. Schrack, L. Kaestner, M. W. Laschke, C. Wagner, S. Gekle, Antimargination of microparticles and platelets in the vicinity of branching vessels. *Biophys. J.* **115**, 411–425 (2018).

51. E. Evans, Y.-C. Fung, Improved measurements of the erythrocyte geometry. *Microvasc. Res.* **4**, 335–347 (1972).

52. P. Balogh, P. Bagchi, A computational approach to modeling cellular-scale blood flow in complex geometry. *J. Comput. Phys.* **334**, 280–307 (2017).

53. R. Mittal, H. Dong, M. Bozkurttas, F. Najjar, A. Vargas, A. Von Loebbecke, A versatile sharp interface immersed boundary method for incompressible flows with complex boundaries. *J. Comput. Phys.* **227**, 4825–4852 (2008).

54. S. M. Recktenwald, K. Graessel, F. M. Maurer, T. John, S. Gekle, C. Wagner, Red blood cell shape transitions and dynamics in time-dependent capillary flows. *Biophys. J.* **121**, 23–36 (2022).

55. M. Nouaman, A. Darras, T. John, G. Simionato, M. A. Rab, R. van Wijk, M. W. Laschke, L. Kaestner, C. Wagner, S. M. Recktenwald, Effect of cell age and membrane rigidity on red blood cell shape in capillary flow. *Cell* **12**, 1529 (2023).

56. X. Zhang, W. A. Lam, M. D. Graham, Dynamics of deformable straight and curved prolate capsules in simple shear flow. *Phys. Rev. Fluids* **4**, 043103 (2019).

57. D. A. Reasor Jr, M. Mehrabadi, D. N. Ku, C. K. Aidun, Determination of critical parameters in platelet margination. *Ann. Biomed. Eng.* **41**, 238–249 (2013).

58. D. Kaul, D. Chen, J. Zhan, Adhesion of sickle cells to vascular endothelium is critically dependent on changes in density and shape of the cells. *Blood* **83**, 3006–3017 (1994).

59. G. Nash, C. Johnson, H. Meiselman, Mechanical properties of oxygenated red blood cells in sickle cell (HbSS) disease. *Blood* **63**, 73–82 (1984).

60. S. Reitsma, D. W. Slaaf, H. Vink, M. A. Van Zandvoort, M. G. oude Egbrink, The endothelial glycocalyx: Composition, functions, and visualization. *Pflugers Arch.* **454**, 345–359 (2007).

61. G. Dai, S. Vaughn, Y. Zhang, E. T. Wang, G. Garcia-Cardena, M. A. Gimbrone Jr., Biomechanical forces in atherosclerosis-resistant vascular regions regulate endothelial redox balance via phosphoinositol 3-kinase/Akt-dependent activation of Nrf2. *Circ. Res.* **101**, 723–733 (2007).

62. S. R. McSweeney, E. Warabi, R. C. Siow, Nrf2 as an endothelial mechanosensitive transcription factor: Going with the flow. *Hypertension* **67**, 20–29 (2016).

63. H. Flyvbjerg, H. G. Petersen, Error estimates on averages of correlated data. *J. Chem. Phys.* **91**, 461–466 (1989).

64. C. Geuzaine, J.-F. Remacle, Gmsh: A 3-D finite element mesh generator with built-in pre-and post-processing facilities. *Int. J. Numer. Methods Eng.* **79**, 1309–1331 (2009).

65. K. C. Ong, M.-C. Lai, Y. Seol, An immersed boundary projection method for incompressible interface simulations in 3D flows. *J. Comput. Phys.* **430**, 110090 (2021).

66. P. B. Canham, The minimum energy of bending as a possible explanation of the biconcave shape of the human red blood cell. *J. Theor. Biol.* **26**, 61–81 (1970).

67. W. Helfrich, Elastic properties of lipid bilayers: Theory and possible experiments. *Z. Naturforsch. C* **28**, 693–703 (1973).

68. R. Skalak, A. Tozeren, R. Zarda, S. Chien, Strain energy function of red blood cell membranes. *Biophys. J.* **13**, 245–264 (1973).

69. G. Tryggvason, B. Bunner, A. Esmaeeli, D. Juric, N. Al-Rawahi, W. Tauber, J. Han, S. Nas, Y.-J. Jan, A front-tracking method for the computations of multiphase flow. *J. Comput. Phys.* **169**, 708–759 (2001).

70. J. B. Freund, J. Vermot, The wall-stress footprint of blood cells flowing in microvessels. *Biophys. J.* **106**, 752–762 (2014).

71. P. Balogh, P. Bagchi, Three-dimensional distribution of wall shear stress and its gradient in red cell-resolved computational modeling of blood flow in in vivo-like microvascular networks. *Physiol. Rep.* **7**, e14067 (2019).

72. D.-V. Le, S. T. Wong, A front-tracking method with Catmull–Clark subdivision surfaces for studying liquid capsules enclosed by thin shells in shear flow. *J. Comput. Phys.* **230**, 3538–3555 (2011).

73. R. Fåhræus, T. Lindqvist, The viscosity of the blood in narrow capillary tubes. *Am. J. Physiol.* **96**, 562–568 (1931).

74. A. R. Pries, D. Neuhaus, P. Gaehtgens, Blood viscosity in tube flow: Dependence on diameter and hematocrit. *Am. J. Physiol.* **263**, H1770–H1778 (1992).

75. Y. Fung, B. Zweifach, Microcirculation: Mechanics of blood flow in capillaries. *Annu. Rev. Fluid Mech.* **3**, 189–210 (1971).

76. A. Pries, K. Ley, M. Claassen, P. Gaehtgens, Red cell distribution at microvascular bifurcations. *Microvasc. Res.* **38**, 81–101 (1989).

77. C. Dupont, A.-V. Salsac, D. Barthès-Biesel, Off-plane motion of a prolate capsule in shear flow. *J. Fluid Mech.* **721**, 180–198 (2013).

78. Z. Wang, Y. Sui, P. D. Spelt, W. Wang, Three-dimensional dynamics of oblate and prolate capsules in shear flow. *Phys. Rev. E Stat. Nonlin. Soft Matter. Phys.* **88**, 053021 (2013).

79. L. Lanotte, J. Mauer, S. Mendez, D. A. Fedosov, J.-M. Fromental, V. Claveria, F. Nicoud, G. Gompper, M. Abkarian, Red cells' dynamic morphologies govern blood shear thinning under microcirculatory flow conditions. *Proc. Natl. Acad. Sci. U.S.A.* **113**, 13289–13294 (2016).

80. C. Minetti, V. Audemar, T. Podgorski, G. Coupier, Dynamics of a large population of red blood cells under shear flow. *J. Fluid Mech.* **864**, 408–448 (2019).

81. D. Cordasco, P. Bagchi, Orbital drift of capsules and red blood cells in shear flow. *Phys. Fluids* **25**, 091902 (2013).

82. M. Bitbol, Red blood cell orientation in orbit C = 0. *Biophys. J.* **49**, 1055–1068 (1986).

83. Z. Peng, A. Mashayekh, Q. Zhu, Erythrocyte responses in low-shear-rate flows: Effects of non-biconcave stress-free state in the cytoskeleton. *J. Fluid Mech.* **742**, 96–118 (2014).

84. D. Cordasco, A. Yazdani, P. Bagchi, Comparison of erythrocyte dynamics in shear flow under different stress-free configurations. *Phys. Fluids* **26**, 041902 (2014).

85. J. Mauer, S. Mendez, L. Lanotte, F. Nicoud, M. Abkarian, G. Gompper, D. A. Fedosov, Flow-induced transitions of red blood cell shapes under shear. *Phys. Rev. Lett.* **121**, 118103 (2018).

86. M. Abkarian, M. Faivre, A. Viallat, Swinging of red blood cells under shear flow. *Phys. Rev. Lett.* **98**, 188302 (2007).

87. F. Reichel, J. Mauer, A. A. Nawaz, G. Gompper, J. Guck, D. A. Fedosov, High-throughput microfluidic characterization of erythrocyte shapes and mechanical variability. *Biophys. J.* **117**, 14–24 (2019).
